# Isolation, antibiogram and whole genome sequence analysis of *Pasteurella multocida* type A from layer birds

**DOI:** 10.1371/journal.pone.0357005

**Published:** 2026-08-31

**Authors:** Md Ahosanul Haque Shahid, Ajran Kabir, Shanta Das, Md. Tanvir Rahman, K. H. M. Nazmul Hussain Nazir

**Affiliations:** Department of Microbiology and Hygiene, Faculty of Veterinary Science, Bangladesh Agricultural University, Mymensingh, Bangladesh; MOHME: Iran Ministry of Health and Medical Education, IRAN, ISLAMIC REPUBLIC OF

## Abstract

Fowl cholera (FC), caused by *Pasteurella multocida* type A, represents a major threat to poultry production worldwide. Detailed characterization of the genotypic, phenotypic, and antimicrobial resistance profiles is essential for advancing epidemiological surveillance and guiding effective intervention strategies. A total of 80 suspected FC field samples were collected from which *P. multocida* was isolated and identified through conventional culture techniques, Gram staining, and PCR. Antimicrobial susceptibility testing was performed using the disc diffusion method with 16 antimicrobial drugs. Whole-genome sequencing of a representative isolate was conducted using the Illumina MiSeq platform, and genomic analysis was carried out with multiple bioinformatics tools. *P. multocida* type A was confirmed in 12 isolates (15%). RAST annotation revealed a genome size of 2,346,689 bp with 2,205 predicted coding sequences, 59 RNA genes, and a GC content of 40.2%. Phenotypic analyses showed resistance to cefixime, ceftriaxone, ertapenem, and meropenem, while the highest sensitivity was observed for ciprofloxacin, doxycycline, tetracycline, and levofloxacin. PathogenFinder predicted the isolate as a potential human pathogen with a probability score of 0.891. Virulence-associated genes identified included *wecA, galU, manB, rfaD, rfaE, rfaF, lpxB, lpxC,* and *msbA*, supporting the pathogenic potential of the isolates and suggesting a possible role in host immune evasion. Phylogenetic analysis using MEGA X with the maximum likelihood method revealed that the isolate was clustered within the monophyletic clade and that it showed closest genetic relatedness to strains from Australia, China, and Malaysia. These findings highlight the genomic basis of virulence and antimicrobial resistance in *P. multocida* type A from Bangladeshi layer for the first time. The isolate characterized in this study provides valuable genomic insights and the selection of targeted antimicrobials to improve fowl cholera control strategies.

## 1. Introduction

Commercial poultry farming in Bangladesh began in the 1980s and has expanded rapidly in recent decades, driven by the intensive rearing of high-yielding chick strains. The sector plays a pivotal role in improving human nutrition through the supply of meat and eggs, while also contributing to income generation and poverty alleviation. Currently, more than 150,000 commercial farms, with an estimated investment of 350 thousand million Bangladeshi Taka, support the livelihoods of approximately six to eight million people in Bangladesh [1].

Despite favorable agro-climatic conditions, the growing poultry industry in Bangladesh faces recurrent outbreaks of infectious and noninfectious diseases, leading to weight loss, reduced egg production, and elevated mortality, and therefore considerable economic losses [1]. Among bacterial diseases, Fowl Cholera (FC) is a critical threat, significantly hampering poultry production [2]. FC is an acute septicemic disease with high morbidity and mortality in chickens and is prevalent in 25–35% of Bangladeshi poultry populations, with higher rates in backyard flocks (59–72%) compared to layers (12.5%) and broilers (4.25%) [3–5]. The disease is caused by *P. multocida*, a commensal bacteria species of the upper respiratory tract that can also induce acute septicemia in cattle, sheep, and goats, resulting in heavy economic losses [6,7]. Based on capsular antigens, *P. multocida* is divided into five capsular types (A, B, D, E, F) and 16 serovars based on lipopolysaccharide antigens [8]. In Asian countries, *P. multocida* types A:1, A:3, and D are commonly associated with FC, with serovar A:1 causing up to 80% mortality [9]. Layers are particularly susceptible compared to younger chickens, making the disease especially problematic in egg-producing flocks [10].

Traditional approaches for pathogen characterization, including phenotypic and molecular methods as well as antimicrobial susceptibility testing, are routinely used to manage outbreaks. However, these approaches have limitations such as low genomic resolution and the target-specific scope of molecular assays [11,12]. Conventional genotypic tools frequently detect antimicrobial resistance (AMR) genes but not virulence determinants, thereby limiting their utility for comprehensive outbreak investigations [13]. In contrast, whole-genome sequencing (WGS) provides high-resolution information on virulence, resistance, host adaptation, and evolutionary relationships. Advances in sequencing platforms and bioinformatics pipelines have reduced costs while increasing speed and accuracy [14,15]. Numerous studies now demonstrate that WGS offers a rapid, cost-effective, and superior method for outbreak analysis compared with traditional approaches [16–19].

In Bangladesh, although phenotypic methods are widely used to characterize *P. multocida*, genetic information is rarely integrated into outbreak management. Some locally produced vaccines are applied in the field, but vaccination failures remain common, likely due to inadequate genomic insights into circulating strains [10,20,21]. To date, no whole-genome sequence data are available for *P. multocida* type A isolates from layer hens in Bangladesh. Therefore, this study was undertaken to isolate and identify *P. multocida* from recent field cases of fowl cholera in layer hens using cultural, biochemical, and molecular approaches; to determine and screening its antimicrobial susceptibility profile by disc diffusion, and to elucidate its genomic features through next-generation sequencing.

## 2. Materials and methods

### 2.1. Study areas and collection of samples

Samples were collected from commercial layer farms located in Trishal (24.5816°N, 90.3948°E, n = 5 farm), Valuka (24.4078°N, 90.3865°E, n = 5 farm), and Gazipur (25.6135°N, 83.5070°E, n = 5 farm). Each farm housed more than 1,000 hens, and at least five hens were sampled per farm. In total, 80 layer hens, each including heart, liver, and spleen tissues, were obtained from layer hens that had died with clinical suspicion of fowl cholera. Most hens were in mid- to late-laying age. According to farm owners, routine vaccination was practiced and flock management followed Department of Livestock Services (Bangladesh) guidelines for feeding and lighting. As these were private commercial farms, detailed information on flock size, production performance, or concurrent disease events was not disclosed. As the birds had either already died or were clinically ill and had been brought to the Veterinary Diagnostic Center for diagnosis, sampling was conducted by a registered veterinarian with the consent of the farm owners and transported to the Department of Microbiology and Hygiene, Bangladesh Agricultural University (BAU), Mymensingh, in an icebox under cold-chain conditions for subsequent bacterial isolation. Therefore, no additional ethical approval was required for this study.

### 2.2. Isolation and Identification through cultural and biochemical examination

Each tissue sample (5–10 g of infected organ) was inoculated into fresh nutrient broth (Himedia, India) and incubated aerobically at 37 °C for 24 h in a bacteriological incubator. Growth-positive broths were streaked onto nutrient agar (Himedia, India) and incubated at 37 °C for 24 h. Suspected *Pasteurella* colonies were subcultured on 5% bovine blood agar (Himedia, India) for enrichment and streaked onto MacConkey agar (Himedia, India) for further confirmation. Representative colonies were examined morphologically by Gram staining and microscopy under oil immersion (100X), and pure isolates were obtained for downstream analysis.

Biochemical characterization of the isolates was performed using a panel of tests, including methyl red–Voges Proskauer (MR-VP), indole, catalase, oxidase, and carbohydrate fermentation (dextrose, maltose, lactose, mannitol, and sucrose) [5]. For preservation, pure isolates were maintained either in 70% glycerol stocks or on blood agar slants at refrigerated conditions to ensure retention of original characteristics.

### 2.3. DNA extraction and molecular detection by PCR

A pure *P. multocida* colony was suspended in 200 μL of deionized water, subjected to heat lysis at 94 °C for 10 min, followed by cold shock on ice for 10 min. The lysate was centrifuged at 10,000 rpm for 10 min, and the supernatant was used as the DNA template [22]. PCR assays targeting the ***KMT1*** and ***capA*** genes were performed to confirm *P. multocida* type A. The primers used in this study are listed in Table 1. Each 25 μL PCR reaction consisted of 12.5 μL 2X PCR Master Mix (GoTaq® G2 Green Master Mix, Promega, USA), 1 μL of each forward and reverse primer (10 μM), 2 μL of DNA template, and 8.5 μL of nuclease-free water [23].

**Table 1:** List of primers used for the detection of *P. multocida* type A.

Specificity	Primer	Sequence (5’-3’)	Amplicon size (bp)	Reference
*P. multocida*	KMT1T7	ATC-CGC-TAT-TTA-CCC-AGT-GG	460 bp	[21]
	KMT1SP6	GCT-GTA-AAC-GAA-CTC-GCC-AC		
*P. multocida* type A	PMcapA F	TGC-CCA-AAT-CGC-AGT-CAG	1044 bp	
	PMcapA R	TTG-CCA-TCA-TTG-TCA-GTG		

F=Forward; R=Reverse; bp=Base Pair.

The thermal cycling profile for the ***KMT1*** gene included: initial denaturation at 94 °C for 5 min; 30 cycles of denaturation at 94 °C for 1 min, annealing at 50 °C for 1 min, and extension at 72 °C for 1 min; followed by a final extension at 72 °C for 9 min, and a 4 °C hold. For the ***capA*** gene, the profile was: initial denaturation at 95 °C for 5 min; 30 cycles of denaturation at 95 °C for 30 s, annealing at 50 °C for 30 s, and extension at 72 °C for 1 min 30 s; followed by a final extension at 72 °C for 5 min, and a 4 °C hold.

PCR products were resolved by electrophoresis on a 1.5% agarose gel (Sigma) at 100 V for 30 min, stained with ethidium bromide (0.5 μg/mL) for 10 min, and destained in distilled water for 10 min. Gels were visualized and documented using a UV Solo transillumination (Biometra, Germany) [24,25].

### 2.4. Antimicrobial sensitivity test

The antimicrobial susceptibility profile of the isolates was determined using a panel of commercially available antimicrobial discs (Himedia, India) selected for antimicrobial resistance surveillance and comparison with previous studies. The antimicrobial tested were ampicillin (AMP, 10 μg); cefixime (CFM, 5 μg); ceftriaxone (CTR, 30 μg); chloramphenicol (C, 30 μg); ciprofloxacin (CIP, 5 μg); colistin (CL, 10 μg); doxycycline (DO, 30 μg); enrofloxacin (ENR, 5 μg); erythromycin (E, 15 μg); ertapenem (ETP, 10 μg); gentamycin (GEN, 10 μg); levofloxacin (LEV, 5 μg); meropenem (MEM, 10 µg); neomycin (N, 30 µg); norfloxacin (NOR, 10 µg); tetracycline (TE, 30 µg). Zone diameters were measured and interpreted according to Clinical and Laboratory Standards Institute [26] and European Committee on Antimicrobial Susceptibility Testing [27] guidelines breakpoints. Isolates were classified as sensitive, intermediate, or resistant.

### 2.5. Whole-genome sequence and data analysis

Based on PCR and antimicrobial sensitivity pattern, one representative isolate (PM58 or BAUFCTA) which showed the most antimicrobial resistance was selected for whole-genome sequencing through Illumina MiSeq Sequencer (NSU, Dhaka) [28]. DNA was extracted by Wizard genomic DNA extraction kit (Promega, USA) according to the manufacturer’s instructions. The sequenced file was assembled by SPAdes 3.9 [29] and annotated with RAST server [30]. Annotated data were analyzed using different bioinformatic tools such as The Comprehensive Antibiotic Resistance Database (CARD) for identification of resistance genes [31] (http://card.mcmaster.ca), Virulence Finder Data Base (https://www.mgc.ac.cn/VFs/) to identify virulence genes, SpeciesFinder 2.0 [32] (https://cge.food.dtu.dk/services/SpeciesFinder/) for species identification, and Pathogenfinder (Version 2) to identify gene families that (https://genepi.food.dtu.dk/pathogenfinder) [33] correlate with pathogenicity. Core-genome alignment was generated using Roary v3.11.2, based on genome annotations produced with Prokka v1.14.5 (https://github.com/tseemann/prokka). The phylogenetic tree was constructed using the maximum likelihood method by CLC Genomics Workbench (Qiagen) with the bootstrap value of 1000 [34].

## 3. Results

### 3.1. Isolation and molecular detection

**Fig 1:**
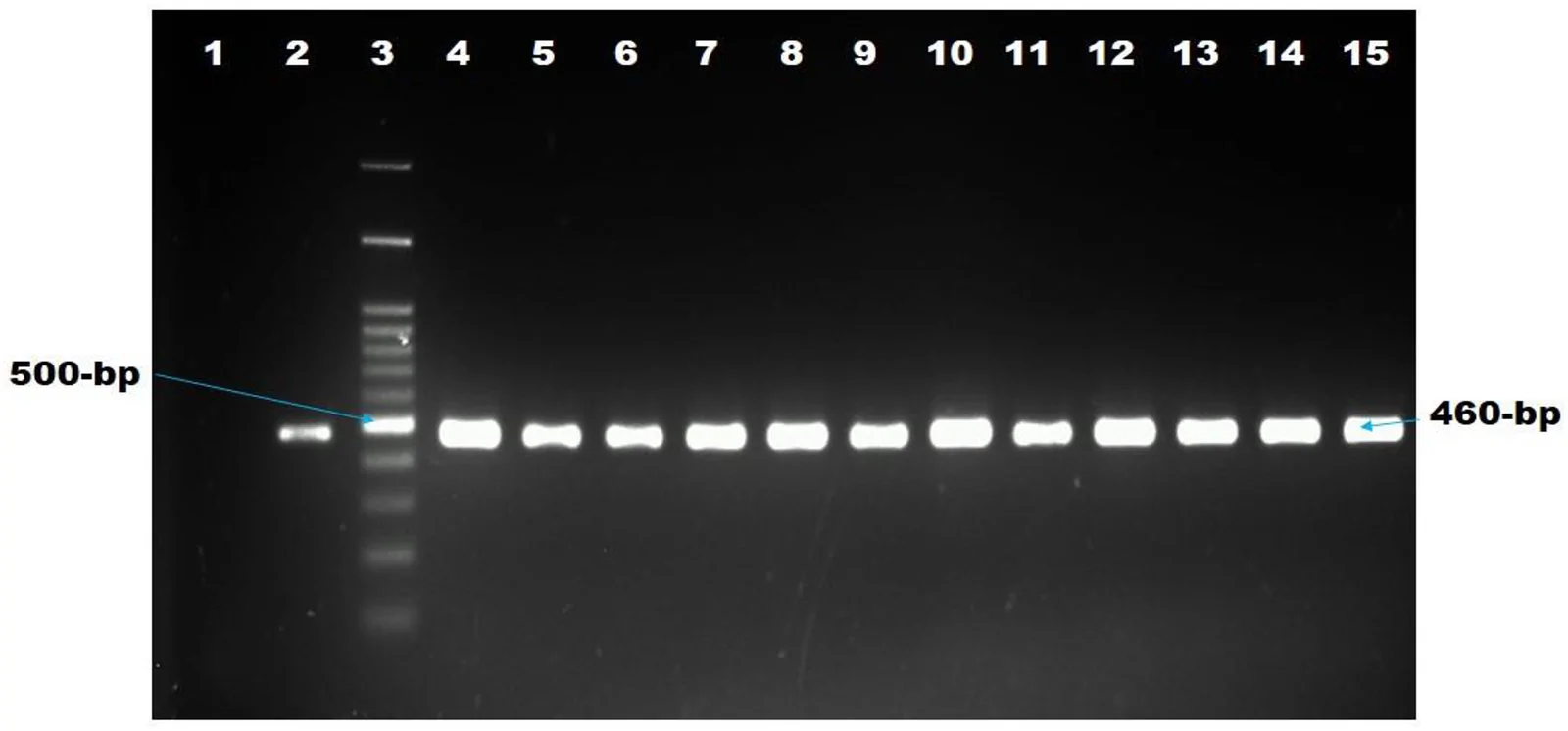
Amplification of *P. multocida* species specific gene (*KMT1*). Lane 1, 2: Negative and Positive Control; Lane 3: 100-bp DNA ladder; Lane 4-15: Representative *P. multocida* isolates.

**Fig 2:**
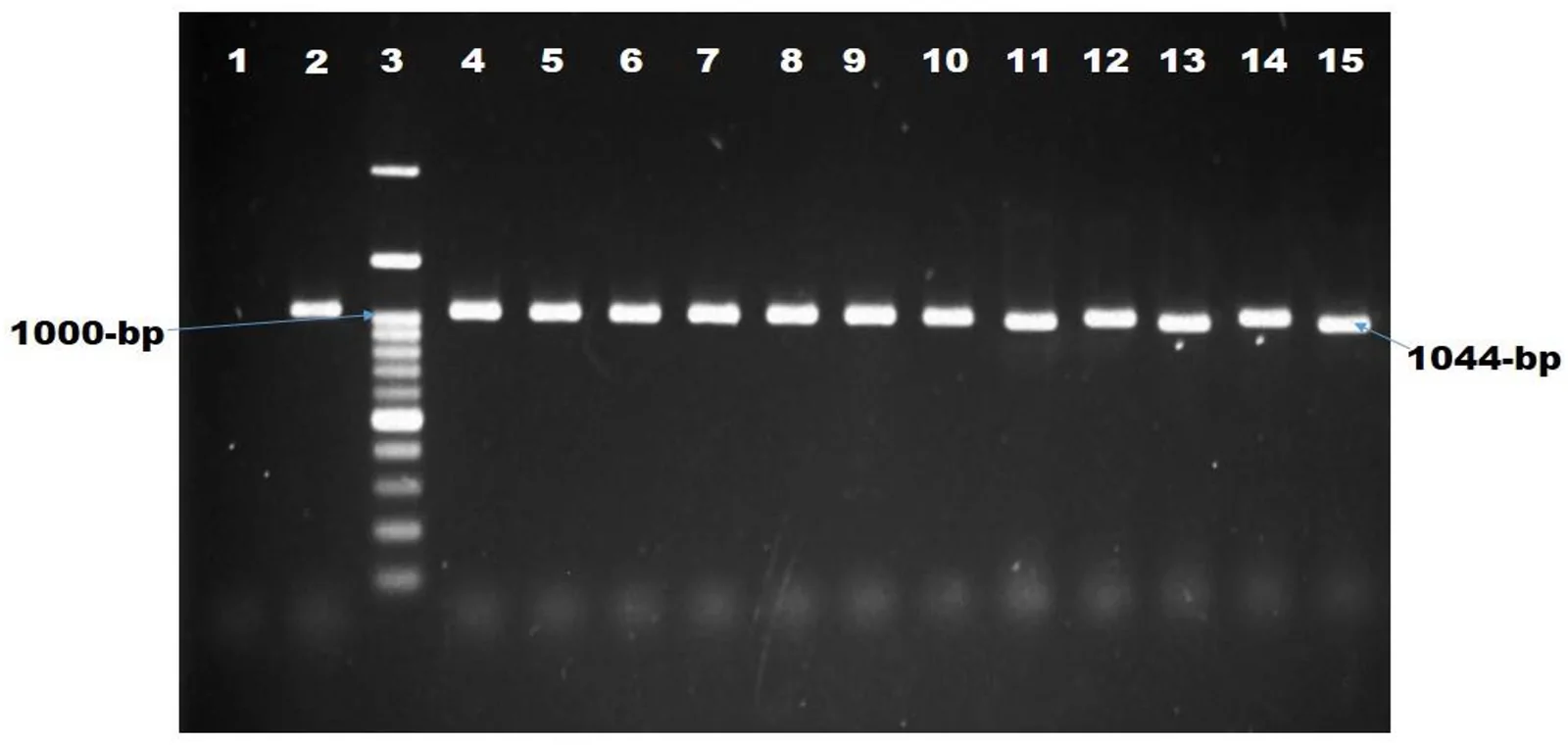
Amplification of *P. multocida* type A specific gene (*capA*). Lane 1, 2: Negative and Positive Control; Lane 3: 100-bp DNA ladder; Lane 4-15: Representative *P. multocida* isolates.

In the present study, 80 suspected FC cases from commercial layer farms were examined. On nutrient agar, the isolates produced spherical, smooth, opaque, white, and round colonies. On 5% bovine blood agar, colonies were small, round, opaque, and non-hemolytic, with a characteristic musty odor, while no growth was observed on MacConkey agar. Microscopically, the organisms appeared as Gram-negative coccobacilli occurring singly or in pairs. Biochemical characterization showed fermentation of dextrose, sucrose, and mannitol without gas production, but no fermentation of lactose or maltose. The isolates were positive for indole, catalase, and oxidase, and negative for methyl red and Voges-Proskauer tests. Samples passed the cultural and biochemical properties used for molecular detection by PCR. Overall *P. multocida* type A was confirmed in 15% (12/80) of cases based on cultural, biochemical, and PCR confirmation of *KMT1* and *capA* genes (Fig 1 and Fig 2).

### 3.2. Antimicrobial sensitivity test

**Fig 3:**
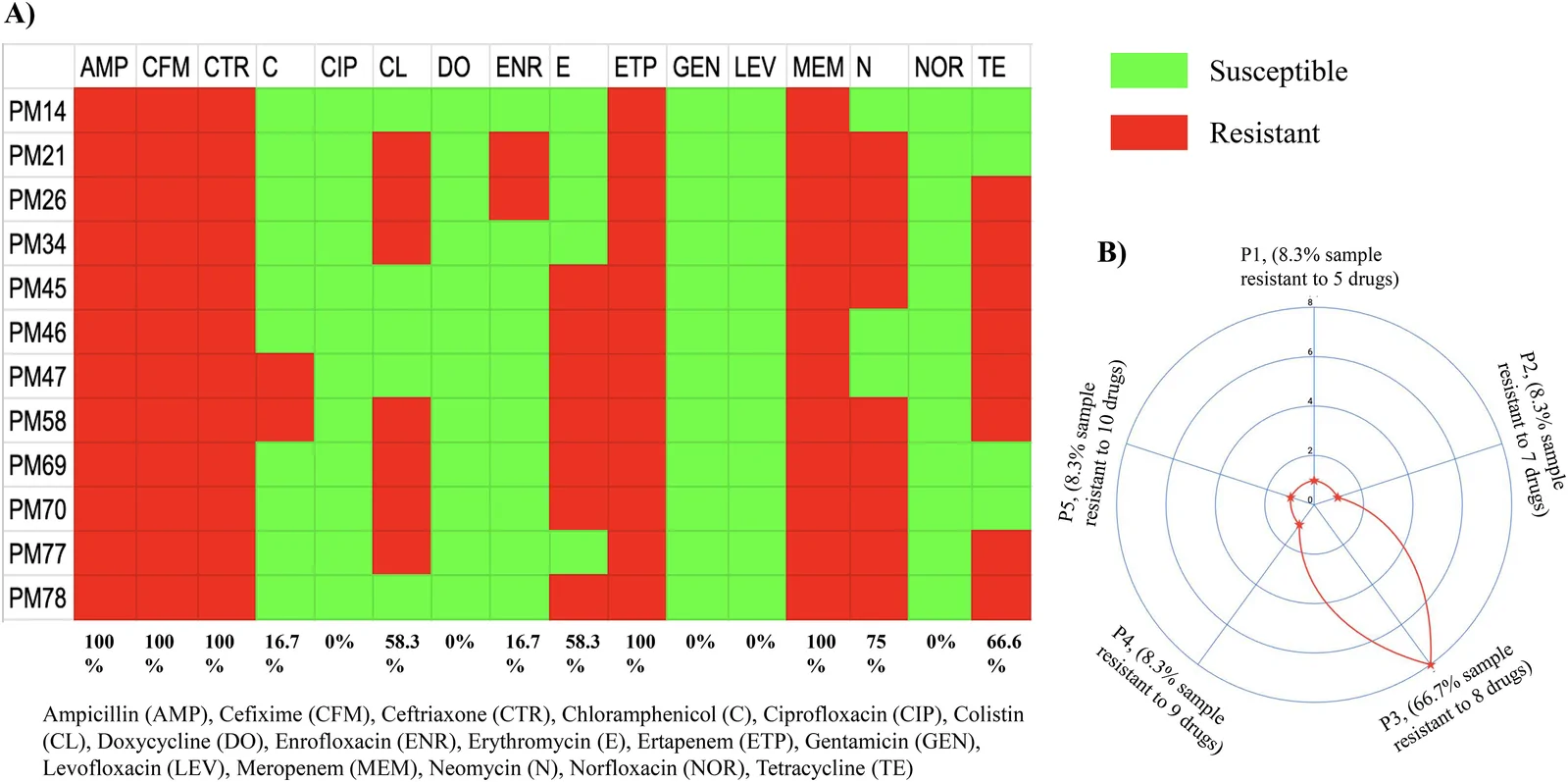
A) The resistance profile of each *P. multocida* isolate against the tested antibiotics along with the number and percentage of isolates resistant to each antimicrobial, and B) the multidrug resistance (MDR) patterns observed among the isolates.

The antimicrobial resistance profiles of the 12 *P. multocida* type A isolates are summarized in Fig 3. All isolates (100%) were resistant to ampicillin, cefixime, ceftriaxone, ertapenem, and meropenem. Resistance was also observed for neomycin (75.0%, 9/12), tetracycline (66.7%, 8/12), colistin (58.3%, 7/12), and erythromycin (58.3%, 7/12). Lower resistance frequencies were detected for chloramphenicol (16.7%, 2/12) and enrofloxacin (16.7%, 2/12), whereas all isolates remained susceptible to ciprofloxacin, doxycycline, gentamicin, levofloxacin, and norfloxacin (Fig 3A).

Analysis of multidrug resistance (MDR) patterns revealed five distinct resistance profiles among the isolates (Fig 3B). The predominant pattern (P3) was observed in 66.7% (8/12) of isolates, each showing resistance to eight antimicrobial agents. The remaining four MDR patterns (P1, P2, P4, and P5) were each detected in 8.3% (1/12) of the isolates, exhibiting resistance to 5, 7, 9, and 10 antimicrobial agents, respectively. These findings demonstrate the predominance of a common MDR phenotype among the *P. multocida* isolates examined.

### 3.3. Genomic features

The isolate PM58, which exhibited the highest level of antimicrobial resistance, was selected for whole-genome sequencing and subsequent analyses. Genome annotation using the RAST server provided an overview of the genome’s taxonomy ID, domain, taxonomy, list of closest neighbors, genome size, GC content percentage, the genome’s N50 and L50 (statistics of a set of contigs or scaffold length), number of contigs (with PEGs), number of subsystems, number of coding sequences and number of RNAs which are listed in Table 2. SpeciesFinder server finds out the strain based on 16s RNA. Here, we can see that the BAUFCTA was identified as *P. multocida* Strain HN06.

**Table 2:** Genome assembly and annotation statistics of BAUFCTA.

Genome	*Pasteurella multocida*
Domain	Bacteria
Taxonomy	Bacteria: *Pasteurella multocida*
Size	2,346,689-bp
GC Content	40.2%
N50	229570
L50	4
Number of Contigs (with PEGs)	82
Number of Subsystems	422
Number of Coding Sequences	2205
Number of RNAs	59

**Fig 4:**
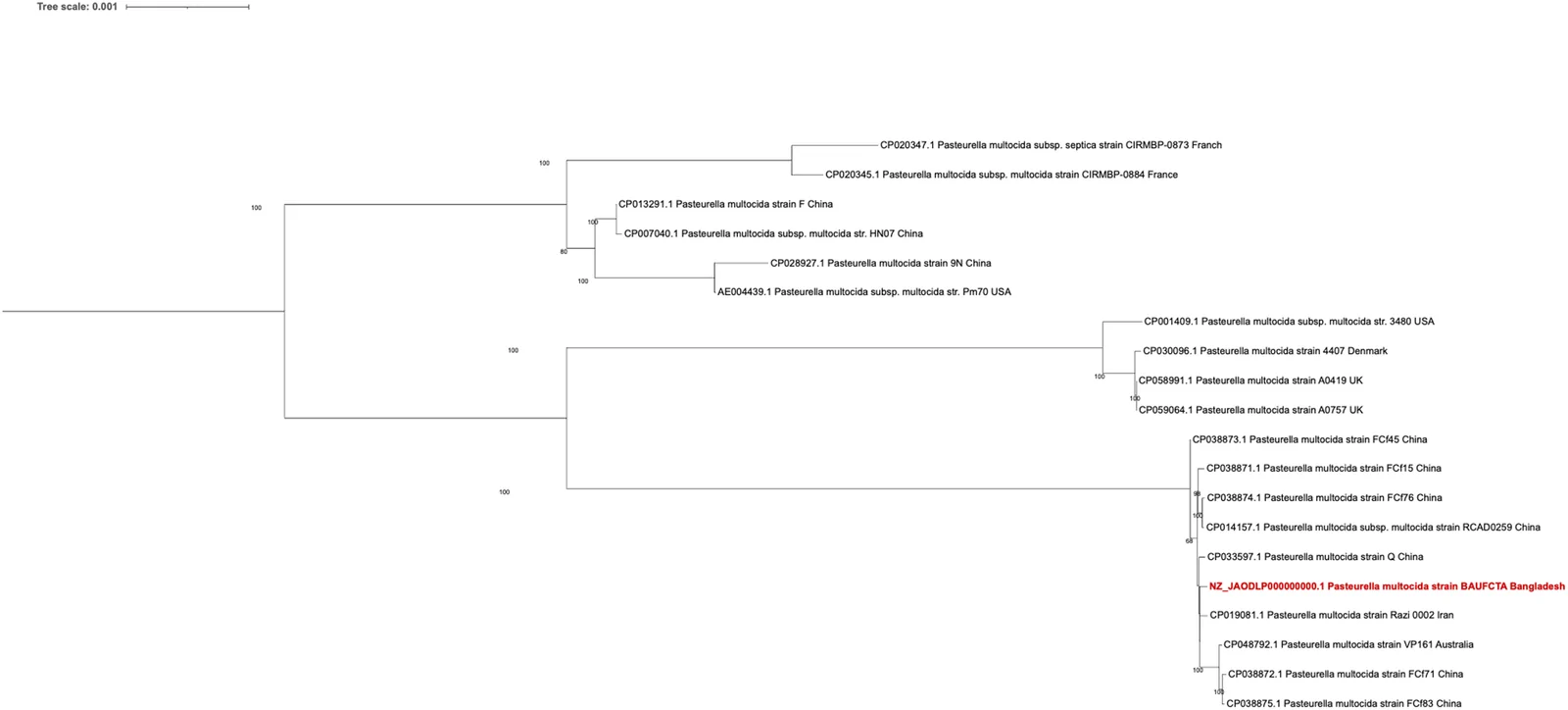
Maximum-likelihood core-genome phylogenetic tree showing the evolutionary relationship of the Bangladeshi *P. multocida* isolate BAUFCTA (highlighted in red) with selected publicly available reference genomes. The tree was constructed using CLC Genomics Workbench v22 based on core-genome alignment with 1,000 bootstrap replicates. Bootstrap values are indicated at the corresponding nodes.

In the distance tree analysis (S2 Fig), the query isolate BAUFCTA (highlighted in yellow) clustered within a monophyletic clade containing closely related *P. multocida* reference genomes, supporting its taxonomic assignment as *P. multocida*. Core genome phylogenetic analysis showed that the Bangladeshi isolate BAUFCTA (highlighted in red) clusters with several international reference strains, forming a strongly supported lineage (Fig 4). PathogenFinder server predicted the BAUFCTA genome as Human pathogenic, and its probability was 0.891.

The SEED Viewer estimates the proportion of genes assigned to subsystems and categorizes them according to functional roles. We have given (S3 Fig) the subsystem feature counts for the BAUFCTA genome. Using Virulence Factor Data Base *P. multocida* BAUFCTA strain was found to harbor multiple virulence-associated genes involved in lipopolysaccharide (LPS) biosynthesis, modification, and export (Table 3). Genomic analysis also identified multiple AMR determinants, including *CRP* (efflux regulation), *tufA* (elfamycin), *uhpT* (fosfomycin), and *parC* (fluoroquinolone) (S1 Table).

**Table 3:** Genome‑wide distribution of virulence factors‑associated genes, and their features in BAUFCTA strain.

Gene	Product	Function	Query Coverage	Identity
*wecA*	Undecaprenyl-phosphate alpha-N-acetylglucosaminyl 1-phosphate transferase (EC 2.7.8.33)	WecA is an integral membrane protein that initiates the biosynthesis of enterobacterial common antigen and O-antigen lipopolysaccharide (LPS).	98	83
*kdsA*	3-deoxy-D-manno-octulosonate-8-phosphate synthase (EC 2.5.1.55)	*kdsA* supplies KDO (3-deoxy-D-manno-oct-2-ulosonic acid) needed to assemble lipopolysaccharide.	94	92
*galU*	UTP--glucose-1-phosphate uridylyltransferase (EC 2.7.7.9)	Makes UDP-glucose, needed for building cell-surface and storage polysaccharides in most bacteria.	96	87
*yhxB* */manB*	Phosphoglucosamine mutase (EC 5.4.2.10)	Encodes the phosphomannomutase that commits mannose into GDP-mannose, a pivotal currency for decorating the bacterial surface.	98	88
*rfaE*	D-glycero-beta-D-manno-heptose 1-phosphate adenylyltransferase (EC 2.7.7.70)/ D-glycero-beta-D-manno-heptose-7-phosphate kinase (EC 2.7.1.167)	Makes ADP-heptose for the lipopolysaccharide inner core.	100	85
*msbB* */LpxM*	Lipid A biosynthesis myristoyltransferase (EC 2.3.1.243)	Caps lipid A with its sixth myristoyl chain; without it, Gram-negative bacteria build a softer, low-immunogenic outer membrane and lose much of their virulence.	80	80
*msbA*	Lipid A export permease/ATP-binding protein msbA	Works as gatekeeper that energizes LPS export: ATP in, lipid A out.	98	80
*lpxC*	UDP-3-O-[3-hydroxymyristoyl] N-acetylglucosamine deacetylase (EC 3.5.1.108)	Zinc-dependent metalloamidase that commits Gram-negative bacteria to lipid A (endotoxin) biosynthesis.	100	91
*lpxB*	Lipid-A-disaccharide synthase (EC 2.4.1.182)	Encodes a glycosyltransferase called lipid A disaccharide synthase	96	81
*rfaD*	ADP-L-glycero-D-manno-heptose-6-epimerase (EC 5.1.3.20)	*rfaD* converts ADP-D,D-heptose into the L-configured form required for lipopolysaccharide core construction.	100	90
*gmhA* */lpcA*	D-sedoheptulose 7-phosphate isomerase (EC 5.3.1.28)	Enzyme that launches the ADP-heptose biosynthetic pathway used to build the inner core of Gram-negative lipopolysaccharide	94	91
*rfaF*	ADP-heptose--lipooligosaccharide heptosyltransferase II	Encodes heptosyl-transferase II, the enzyme that adds the second L-glycero-D-manno-heptose to the inner core of lipopolysaccharide (LPS).	91	83
*galE*	UDP-glucose 4-epimerase (EC 5.1.3.2)	*galE* encodes the epimerase that shuttles UDP-galactose ↔ UDP-glucose, without it, both metabolism and the bacterial cell surface fall apart.	100	86
*orfM* */lpxL*	Nucleoside 5-triphosphatase RdgB (dHAPTP, dITP, XTP-specific) (EC 3.6.1.66)	Works in final steps of lipid A biosynthesis by addition of the fifth acyl chain to lipid A.	97	83

## 4. Discussion

The overall *P. multocida* type A was confirmed 15%. This prevalence is higher than that earlier reported by Hasan et al. [4], who found 12.05% in layer chickens, but lower than rates described in backyard flocks, where prevalence up to 59.72% has been reported [3]. Variations may be explained by differences in flock type, age, breed, season, biosecurity, farm hygiene, nutrition, and vaccine quality. In particular, FC is more frequent in layers at the onset of lay or at older ages [10]. It is well recognized that suboptimal nutrition can impair immune function (e.g., vitamin deficiencies) and that currently available vaccines may provide limited or short-lived protection in poultry [35,36]. Together, these factors may exacerbate susceptibility to *P. multocida* in layer flocks.

SpeciesFinder identified the isolate as *P. multocida* and it has *16s rRNA* gene identity with strain HN06, previously reported in pigs in China [37]. In the distance tree analysis, the query isolate BAUFCTA (S2 Fig) clustered within a monophyletic clade containing closely related *P. multocida* reference genomes, supporting its taxonomic assignment as *P. multocida*. From these analyses, we could confirm the species of this bacterium. Furthermore, the core genome phylogenetic analysis revealed that the Bangladeshi isolate BAUFCTA (Fig 4 shown in red) forms a well-supported branch within a cluster of several international reference strains, including the Chinese isolate *P. multocida* strain Q (CP033597.1) and the Iranian isolate strain Razi 0002 (CP019081.1). The topology also showed that BAUFCTA groups within a broader clade containing isolates such as strain VP161 from Australia (CP048792.1), as well as the Chinese strains FCf71 (CP038872.1) and FCf83 (CP038875.1). Although these genomes originate from diverse geographical regions, the consistently high bootstrap support indicates that BAUFCTA shares a relatively recent evolutionary history with these lineages. PathogenFinder predicted zoonotic potential with a probability of 0.891, highlighting its public health significance. Human pasteurellosis has long been recognized as a zoonotic infection by the World Health Organization and has been associated with puerperal fever, sepsis, meningitis, and rarely, lung abscesses, typically following animal bites, scratches, or respiratory exposure [38]. Therefore, the isolate characterized in this study holds significance not only for poultry health and consumers but also for its potential zoonotic risk, especially as an occupational infection.

In the BAUFCTA genome, the largest subsystem feature counts were observed for Amino Acids and Derivatives (253), Carbohydrates (251), Protein metabolism (233), with 44 subsystems responsible for virulence, disease, and defense. Genes involved in Sialic acid metabolism, Murein hydrolases encoded genes, Peptidoglycan biosynthesis encoded genes, Lysozyme inhibitors, and Multidrug Resistance Efflux Pumps were active in the sequenced genome. The subsystem with the lowest feature count is Dormancy and Sporulation 2. It is noteworthy that motility and chemotaxis had 0 annotated genes which is evidence that the bacteria were non-motile, but we did not further confirm this through a motility test.

Many virulence factors including genes involved in capsule formation, LPS, adhesins and fimbriae, iron-regulated and iron acquisition proteins, hyaluronidase, toxins, and sialic acid metabolism have been reported to contribute to *P. mutocida* pathogenesis [39]. The *wecA* gene initiates O-antigen assembly, while *kdsA*, *galU*, and *yhxB/manB* provide precursors such as KDO, UDP-glucose, and GDP-mannose required for LPS construction [40]. Core biosynthesis genes including *rfaE*, *gmhA/lpcA*, *rfaD*, and *rfaF* generate ADP-heptose for inner-core assembly [41,42]. Lipid A, the immunostimulatory moiety of LPS, is synthesized and remodeled by *lpxC*, *lpxB*, *msbB/LpxM*, and *orfM/lpxL*, ensuring proper acyl chain addition and endotoxic activity [43]. Export of lipid A is mediated by *msbA*, whereas *galE* maintains UDP-galactose/UDP-glucose balance [44].

Antimicrobial susceptibility testing revealed high levels of resistance to cefixime, ceftriaxone, ertapenem, and meropenem. Widespread and often indiscriminate use of antimicrobials in poultry production, particularly tetracyclines for prophylaxis and growth promotion, may contribute to the emergence and dissemination of antimicrobial resistance, as reported previously [45,46]. To further investigate the molecular basis of antimicrobial resistance, the genome was analyzed using three dedicated antimicrobial resistance databases, namely NCBI AMRFinderPlus, CARD, and ResFinder. However, none of these analyses identified acquired resistance genes corresponding to the observed multidrug-resistant phenotype. This genotype–phenotype discordance suggests that the resistance phenotype may result from alternative mechanisms, including chromosomal mutations, altered expression of intrinsic resistance determinants such as efflux systems, changes in membrane permeability, or other resistance mechanisms that are not currently represented in available antimicrobial resistance databases. Although resistance-associated loci such as *parC* and *uhpT* were identified, the presence of these genes alone does not confer resistance, as phenotypic resistance generally depends on specific nucleotide mutations or altered gene function. Likewise, *crp* is a global regulatory protein and *tufA* is a conserved housekeeping gene rather than a confirmed antimicrobial resistance determinant. A comprehensive analysis of resistance-associated single nucleotide polymorphisms (SNPs) and functional validation was beyond the scope of the present study. Therefore, future investigations incorporating mutation analysis and experimental validation will be necessary to elucidate the molecular mechanisms underlying the observed antimicrobial resistance phenotype in *P. multocida*. Just to be clear, carbapenems are not routinely used in poultry production in Bangladesh, carbapenem-resistant Enterobacteriaceae have previously been reported from poultry, humans, and poultry-associated environments in the country, suggesting dissemination of resistance determinants through environmental reservoirs and horizontal gene transfer rather than direct antimicrobial exposure. Likewise, colistin-resistant bacteria carrying *mcr* genes have been detected in Bangladeshi poultry production systems, indicating that resistance to critically important antimicrobials is already present within the One Health interface. In addition, the antimicrobial panel was used only for AMR surveillance and comparison with previous studies, and the results should not be interpreted only as therapeutic recommendations for poultry, particularly for drugs not approved or recommended for use in food-producing animals. However, the combined genomic and phenotypic insights obtained here provide a valuable foundation for future studies in vaccines development and targeted antimicrobial strategies to control fowl cholera (caused by type A) in Bangladesh. We also acknowledge a limitation that due to funding constraints and limited access to local sequencing equipment, we were unable to sequence additional isolates, including those exhibiting multidrug resistance. Nevertheless, we believe this study will build confidence in, and highlight the importance of genomic research in Bangladesh, enabling deeper investigations to inform disease control, support One Health policy, advance sustainable development, and help combat antimicrobial resistance (AMR).

## 5. Conclusion and applications

In this study, 15% (12/80) of samples from Bangladeshi layer hens were confirmed as *P. multocida* type A. Antimicrobial susceptibility testing demonstrated consistent resistance to ampicillin, cefixime, ceftriaxone, ertapenem, and meropenem, with high sensitivity to ciprofloxacin and levofloxacin. Notably, resistance to cefixime, ceftriaxone, ertapenem, and meropenem was supported by both phenotypic assays and some genomic analysis, underscoring the reliability of integrated approaches. Whole-genome sequencing revealed key virulence genes (*wecA, galU, manB, rfaD, rfaE, rfaF, lpxB, lpxC, msbA*) highlighting its pathogenic potential. Phylogenetic analysis clustered the isolate within the monophyletic clade which closely related to Australian, Chinese, and Malaysian strains. PathogenFinder predicted zoonotic potential with a probability score of 0.891, reinforcing its public health relevance specifically for occupational infection. Given the persistence of vaccination failures in Bangladesh, this well-characterized isolate provides a valuable genetic insight to guide targeted interventions for controlling fowl cholera.

## Supporting information

S1 Fig Original uncropped gel images for Fig 1 and Fig 2. (PDF)

S2 Fig Distance tree analysis of BAUFCTA. (PDF)

S3 Fig Subsystem feature counts for the BAUFCTA genome. (JPG)

S1 Table Antimicrobial resistance genes in the genome of BAUFCTA. (DOCX)
