# Supplementary material for: Isolation, antibiogram and whole genome sequence analysis of Pasteurella multocida type A from layer birds
Source: PLoS One. 2026 Aug 31;21(8):e0357005. doi: 10.1371/journal.pone.0357005 (PMC13528996; doi:10.1371/journal.pone.0357005)
Supplement: Supplementary file 1 [file pone.0357005.s001.pdf]

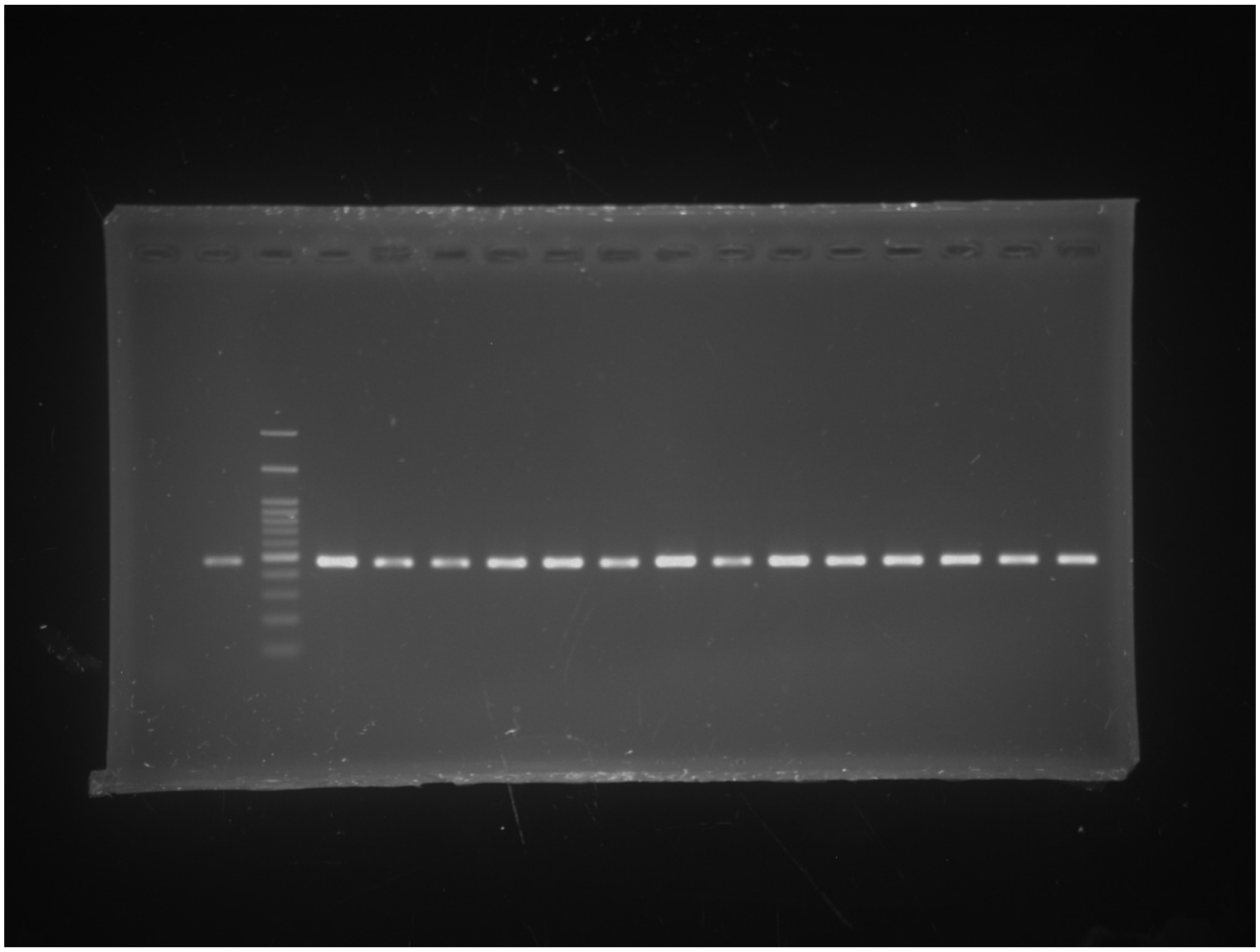

Fig 1. Amplification of *P. multocida* species specific gene *KMT1*

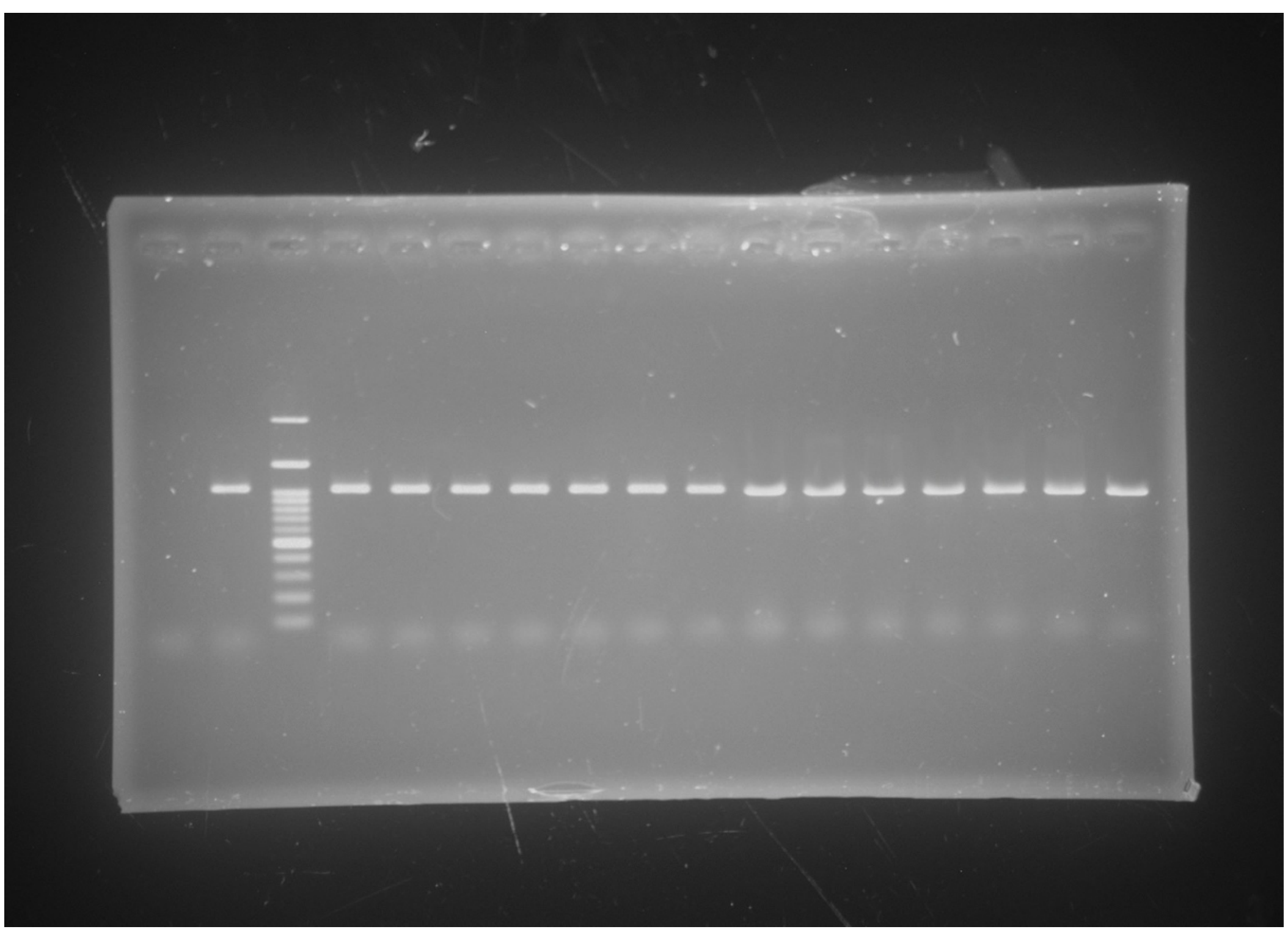

Fig 2. Amplification of *P. multocida* type A specific gene *capA*
