# Supplementary material for: Isolation, antibiogram and whole genome sequence analysis of Pasteurella multocida type A from layer birds
Source: PLoS One. 2026 Aug 31;21(8):e0357005. doi: 10.1371/journal.pone.0357005 (PMC13528996; doi:10.1371/journal.pone.0357005)
Supplement: Supplementary file 2 [file pone.0357005.s002.pdf]

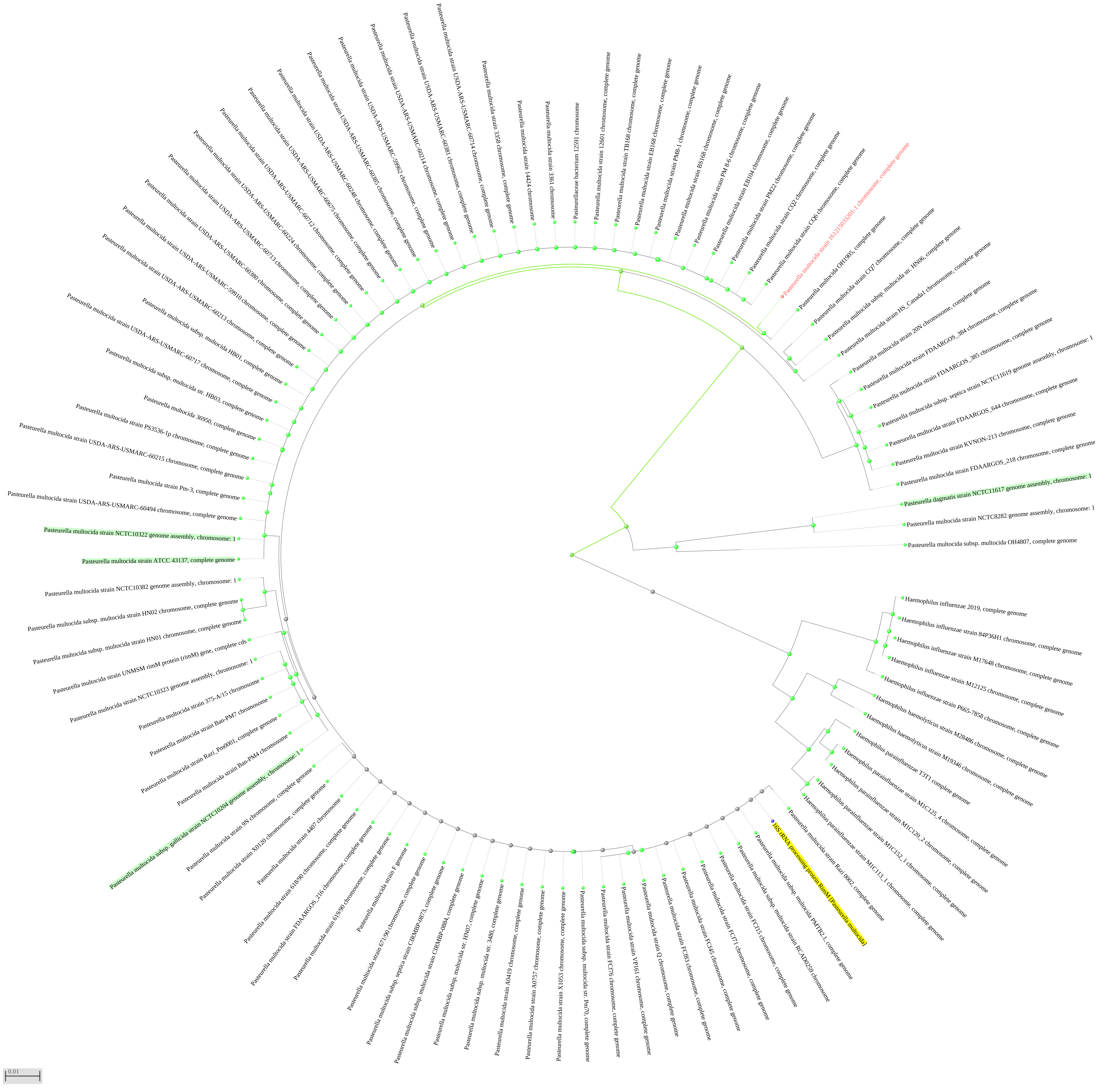
Pasteurella multocida subsp. gallicida strain NCTC10204 genome assembly, chromosome: 1
Pasteurella multocida strain Ban-PM4 chromosome
Pasteurella multocida strain Razi_Pm0001, complete genome
Pasteurella multocida strain Ban-PM7 chromosome
Pasteurella multocida strain 375-A/15 chromosome
Pasteurella multocida strain NCTC10323 genome assembly, chromosome: 1
Pasteurella multocida strain UNMSM rimM protein (rimM) gene, complete cds
Pasteurella multocida subsp. multocida strain HN01 chromosome, complete genome
Pasteurella multocida subsp. multocida strain HN02 chromosome, complete genome
Pasteurella multocida strain NCTC10382 genome assembly, chromosome: 1
Pasteurella multocida strain ATCC 43137, complete genome
Pasteurella multocida strain NCTC10322 genome assembly, chromosome: 1
Pasteurella multocida strain USDA-ARS-USMARC-60494 chromosome, complete genome
Pasteurella multocida strain Pm-3, complete genome
Pasteurella multocida strain USDA-ARS-USMARC-60215 chromosome, complete genome
Pasteurella multocida strain PS3536-1p chromosome, complete genome
Pasteurella multocida 36950, complete genome
Pasteurella multocida subsp. multocida str. HB03, complete genome
Pasteurella multocida strain USDA-ARS-USMARC-60717 chromosome, complete genome
Pasteurella multocida subsp. multocida HB01, complete genome
Pasteurella multocida strain USDA-ARS-USMARC-60213 chromosome, complete genome
Pasteurella multocida strain USDA-ARS-USMARC-59910 chromosome, complete genome
Pasteurella multocida strain USDA-ARS-USMARC-60380 chromosome, complete genome
Pasteurella multocida strain USDA-ARS-USMARC-60713 chromosome, complete genome
Pasteurella multocida strain USDA-ARS-USMARC-60224 chromosome, complete genome
Pasteurella multocida strain USDA-ARS-USMARC-60712 chromosome, complete genome
Pasteurella multocida strain USDA-ARS-USMARC-60675 chromosome, complete genome
Pasteurella multocida strain USDA-ARS-USMARC-60248 chromosome, complete genome
Pasteurella multocida strain USDA-ARS-USMARC-60385 chromosome, complete genome
Pasteurella multocida strain USDA-ARS-USMARC-59962 chromosome, complete genome
Pasteurella multocida strain USDA-ARS-USMARC-60214 chromosome, complete genome
Pasteurella multocida strain USDA-ARS-USMARC-60381 chromosome, complete genome
Pasteurella multocida strain USDA-ARS-USMARC-60714 chromosome, complete genome
Pasteurella multocida strain 3358 chromosome, complete genome
Pasteurella multocida strain 14424 chromosome
Pasteurella multocida strain 3361 chromosome
Pasteurellaceae bacterium 12591 chromosome
Pasteurella multocida strain 12601 chromosome, complete genome
Pasteurella multocida strain TB168 chromosome, complete genome
Pasteurella multocida strain EB168 chromosome, complete genome
Pasteurella multocida strain PM8-1 chromosome, complete genome
Pasteurella multocida strain BS168 chromosome, complete genome
Pasteurella multocida strain PM 8-6 chromosome, complete genome
Pasteurella multocida strain EB104 chromosome, complete genome
Pasteurella multocida strain PM22 chromosome, complete genome
Pasteurella multocida strain CQ2 chromosome, complete genome
Pasteurella multocida strain CQ6 chromosome, complete genome
Pasteurella multocida strain 161215033201-1 chromosome, complete genome
Pasteurella multocida OH1905, complete genome
Pasteurella multocida strain CQ7 chromosome, complete genome
Pasteurella multocida subsp. multocida str. HN06, complete genome
Pasteurella multocida strain HS_Canada1 chromosome, complete genome
Pasteurella multocida strain 20N chromosome, complete genome
Pasteurella multocida strain FDAARGOS_384 chromosome, complete genome
Pasteurella multocida strain FDAARGOS_385 chromosome, complete genome
Pasteurella multocida subsp. septica strain NCTC11619 genome assembly, chromosome: 1
Pasteurella multocida strain FDAARGOS_644 chromosome, complete genome
Pasteurella multocida strain KVNON-213 chromosome, complete genome
Pasteurella multocida strain FDAARGOS_218 chromosome, complete genome
Pasteurella dagmatis strain NCTC11617 genome assembly, chromosome: 1
Pasteurella multocida strain NCTC8282 genome assembly, chromosome: 1
Pasteurella multocida subsp. multocida OH4807, complete genome
Haemophilus influenzae 2019, complete genome
Haemophilus influenzae strain 84P36H1 chromosome, complete genome
Haemophilus influenzae strain M17648 chromosome, complete genome
Haemophilus influenzae strain M12125 chromosome, complete genome
Haemophilus influenzae strain P665-7858 chromosome, complete genome
Haemophilus haemolyticus strain M28486 chromosome, complete genome
Haemophilus haemolyticus strain M19346 chromosome, complete genome
Haemophilus parainfluenzae T3T1 complete genome
Haemophilus parainfluenzae strain M1C125_4 chromosome, complete genome
Haemophilus parainfluenzae strain M1C120_2 chromosome, complete genome
Haemophilus parainfluenzae strain M1C152_1 chromosome, complete genome
Haemophilus parainfluenzae strain M1C113_1 chromosome, complete genome
Pasteurella multocida strain Razi 0002, complete genome
16S rRNA processing protein RimM [Pasteurella multocida]
Pasteurella multocida subsp. multocida PMTB2.1, complete genome
Pasteurella multocida subsp. multocida strain RCAD0259 chromosome
Pasteurella multocida strain FCf15 chromosome, complete genome
Pasteurella multocida strain FCf71 chromosome, complete genome
Pasteurella multocida strain FCf45 chromosome, complete genome
Pasteurella multocida strain FCf83 chromosome, complete genome
Pasteurella multocida strain Q chromosome, complete genome
Pasteurella multocida strain VP161 chromosome, complete genome
Pasteurella multocida strain FCf76 chromosome, complete genome
Pasteurella multocida subsp. multocida str. Pm70, complete genome
Pasteurella multocida strain X1053 chromosome, complete genome
Pasteurella multocida strain A0757 chromosome, complete genome
Pasteurella multocida strain A0419 chromosome, complete genome
Pasteurella multocida subsp. multocida str. 3480, complete genome
Pasteurella multocida subsp. multocida str. HN07, complete genome
Pasteurella multocida subsp. multocida strain CIRMBP-0884, complete genome
Pasteurella multocida subsp. septica strain CIRMBP-0873, complete genome
Pasteurella multocida strain 671/90 chromosome, complete genome
Pasteurella multocida strain F genome
Pasteurella multocida strain 619/90 chromosome, complete genome
Pasteurella multocida strain FDAARGOS_216 chromosome, complete genome
Pasteurella multocida strain 618/90 chromosome, complete genome
Pasteurella multocida strain 4407 chromosome
Pasteurella multocida strain X0120 chromosome, complete genome
Pasteurella multocida strain 9N chromosome, complete genome
0.01
