# Supplementary material for: Isolation, antibiogram and whole genome sequence analysis of Pasteurella multocida type A from layer birds
Source: PLoS One. 2026 Aug 31;21(8):e0357005. doi: 10.1371/journal.pone.0357005 (PMC13528996; doi:10.1371/journal.pone.0357005)
Supplement: Supplementary file 3 [file pone.0357005.s004.docx]

**S1 Table** Antimicrobial resistance genes in the genome of BAUFCTA strain

| Gene | Product | Function | Classification |
| --- | --- | --- | --- |
| *CRP* | Cyclic AMP receptor protein | The CRP gene, encoding the Cyclic AMP Receptor Protein (CRP), plays a complex role in the context of bacterial antimicrobial resistance | Efflux pump conferring antibiotic resistance, gene modulating antibiotic efflux |
| *tufA* | Translation elongation factor Tu | The *tufA* gene encodes for elongation factor Tu. EF-Tu is a critical component of the protein synthesis machinery within bacteria, and its function involves binding to charged transfer RNAs (tRNAs) and delivering them to the ribosome's A-site during translation. | Antibiotic resistant gene variant or mutant, Elfamycin resistance gene |
| *UhpT* | Hexose phosphate transport protein UhpT | Responsible for transporting glucose-6-phosphate (G6P) into bacterial cells. | Antibiotic resistant gene variant or mutant, Fosfomycin resistance gene |
| *parC* | DNA topoisomerase IV subunit A (EC 5.99.1.3) | Encodes a subunit of the enzyme topoisomerase IV | Antibiotic resistant gene variant or mutant, fluoroquinolone resistance gene |
