# Supplementary material for: Isolation, antibiogram and whole genome sequence analysis of Pasteurella multocida type A from layer birds
Source: PLoS One. 2026 Aug 31;21(8):e0357005. doi: 10.1371/journal.pone.0357005 (PMC13528996; doi:10.1371/journal.pone.0357005)
Supplement: Supplementary file 4 [file pone.0357005.s006.docx]

The manuscript addresses an important and timely topic by investigating the antimicrobial susceptibility and whole-genome characteristics of Pasteurella multocida type A isolates recovered from layer chickens in Bangladesh, and the reviewers acknowledge its potential contribution to poultry disease surveillance and antimicrobial resistance monitoring. However, several major concerns must be addressed before the manuscript can be considered for publication.

The most significant limitation is that whole-genome sequencing was performed on only one isolate, while conclusions regarding virulence factors and antimicrobial resistance profiles are generalized to all isolates. In addition, substantial discrepancies exist between the phenotypic antimicrobial susceptibility results and the reported genomic resistance determinants, requiring a more rigorous analysis and interpretation of genotype–phenotype correlations. The presentation and interpretation of virulence genes should be revised to avoid overstatement of their biological significance, and additional genomic analyses (e.g., MLST, capsular/LPS typing, plasmid and mobile genetic element characterization) should be considered or their omission justified. Several figures and tables require substantial revision, simplification, or relocation to supplementary materials, and the rationale for antimicrobial selection, particularly chloramphenicol, carbapenems, and multiple quinolones, should be clearly explained. Furthermore, the reported resistance to carbapenems and colistin warrants additional discussion in the context of antimicrobial usage practices in Bangladesh. Clarification of methodological details, standardization of terminology, inclusion of appropriate statistical analyses where applicable, and careful revision of data presentation and interpretation are also necessary. Overall, while the study has merit and addresses an important veterinary and public health issue, a major revision is required to strengthen its scientific rigor and support the conclusions drawn.

Response: We sincerely thank the Editor for the careful evaluation of our manuscript and for the constructive summary of the major concerns. We appreciate the opportunity to revise our work and have carefully addressed all comments raised by both the Editor and the reviewers. The manuscript has been substantially revised to improve its scientific rigor, clarity, and presentation.

Specifically, we have acknowledged that whole-genome sequencing was performed on a single representative isolate and have clearly stated this as a limitation of the study. We emphasize that the identified virulence-associated genes and genomic characteristics are specific to the sequenced isolate and should not be generalized to all P. multocida isolates. We have also highlighted that additional sequencing and comparative genomic analyses are planned in our ongoing research.

To address the discrepancy between phenotypic antimicrobial susceptibility and genomic resistance determinants, we reanalyzed the genome using three dedicated antimicrobial resistance databases (NCBI AMRFinderPlus, CARD, and ResFinder). No acquired resistance genes corresponding to the observed multidrug-resistant phenotype were identified. Accordingly, we revised both the Results and Discussion to clarify that the observed resistance may result from chromosomal mutations, altered expression of intrinsic resistance mechanisms, changes in membrane permeability, efflux systems, or other mechanisms not represented in current resistance gene databases. We also revised the interpretation of **crp, tufA, uhpT,** and **parC**, making it clear that these genes are not, by themselves, confirmed antimicrobial resistance determinants. Also the table has been removed from the main file.

The presentation and interpretation of virulence-associated genes have also been revised to avoid overstatement. We clearly state that these genes were identified through **in silico** genome annotation and have not been experimentally validated. This limitation has been acknowledged in the manuscript, and future studies involving gene expression and functional validation have been proposed.

The manuscript presentation has been substantially improved. The original antimicrobial susceptibility table has been replaced with a graphical summary illustrating antimicrobial resistance frequencies, isolate-specific resistance patterns, and multidrug resistance (MDR) profiles. Descriptive genome figures have been removed from the main manuscript or transferred to the Supplementary Materials where appropriate. Figure titles, legends, and phylogenetic descriptions have also been revised to better reflect the scope of the analyses.

We have clarified the rationale for the antimicrobial panel used in this study, including the inclusion of chloramphenicol, carbapenems, and multiple fluoroquinolones, emphasizing that these agents were included for epidemiological surveillance and comparison with previous studies rather than as therapeutic recommendations. Furthermore, the Discussion has been expanded to explain the observed resistance to carbapenems and colistin in the context of antimicrobial resistance reports from Bangladesh and the broader One Health framework.

Finally, we carefully revised the manuscript for language, terminology, methodological clarity, and scientific consistency. The terminology has been standardized by consistently using **"antimicrobial"** throughout the manuscript where appropriate, methodological details have been clarified, figure and table presentation has been improved, and the overall discussion has been revised to provide a more balanced interpretation of the findings.

We believe these revisions have substantially strengthened the manuscript and have addressed the concerns raised by both the Editor and the reviewers. We sincerely appreciate the time and effort devoted to evaluating our work and hope that the revised manuscript is now suitable for publication.

Reviewer #1:

Manuscript Title: Isolation, Antibiogram and Whole Genome Sequence Analysis of Pasteurella multocida Type A from Layer Birds
The manuscript presents important findings regarding the isolation, antimicrobial susceptibility, and whole-genome characterization of Pasteurella multocida type A isolated from layer birds in Bangladesh. The integration of phenotypic antimicrobial resistance testing with genomic analysis is valuable and relevant to poultry disease surveillance and antimicrobial resistance monitoring. However, several issues require clarification and revision before the manuscript can be considered for publication.

1. BDT: Please provide the long form in the first mention.

Instead of using short form we used now in full form of BDT.
Response: Thanks for the comment and carefully addressed the issue. [line 49]

2. Line 94: 80-layer hens; from how many farms?

Response: Thanks for asking but farm numbers are already mentioned in line 87-89.
3. Line 139-145: Is chloramphenicol currently approved to be used in veterinary treatment in Bangladesh? If not, justification for its inclusion should be provided. Why testing three quinolones antibiotic discs? I can agree with cephalosporins because they are from different generations! Can you provide justification of using meropenem and ertapenem simultaneously.

Response: Thank you for this important comment. We clarify that this panel was selected for AMR surveillance and comparison with previous regional studies, not as a therapeutic recommendation for poultry. We acknowledge that some antibiotics included in the panel, such as chloramphenicol, meropenem, and ertapenem, are not intended or recommended for use in food-producing animals. Therefore, we have revised the manuscript to clearly state that results for these antibiotics should be interpreted only for epidemiological and One Health AMR monitoring purposes.

Regarding the quinolones/fluoroquinolones, ciprofloxacin, enrofloxacin, levofloxacin, and norfloxacin were included because these drugs are commonly reported in AMR studies and allow comparison of susceptibility patterns within this antimicrobial class. Enrofloxacin is particularly relevant to veterinary use, whereas ciprofloxacin, levofloxacin, and norfloxacin provide comparative surveillance information.

Meropenem and ertapenem were included together to assess whether resistance was consistent across carbapenem agents. However, we agree that these results should not imply clinical use in poultry. We have therefore added a clarification that these drugs were included only for resistance surveillance at the end of discussion. [Line 273-283]

4. Line 148: I'm very concerned with resistance of this pathogen to carbapenems and colistin while susceptible to Fluoroquinolones! Can you provide more explanation about this? Are carbapenems used in poultry production OR veterinary practices in Bangladesh?

**Response:** Thank you for this important comment. We agree that the observed resistance pattern deserves further discussion. To our knowledge, carbapenems are not approved or routinely used in poultry production or veterinary practice in Bangladesh. Therefore, the observed carbapenem resistance is unlikely to result from direct therapeutic use in poultry. However, most recent studies from Bangladesh ([10.1128/spectrum.03956-23](https://doi.org/10.1128/spectrum.03956-23), [10.5455/javar.2019.f311](https://doi.org/10.5455/javar.2019.f311), 10.1038/s41598-020-75608-2) have reported colistin and carbapenem-resistant microbe in poultry, humans, and poultry-associated environments, suggesting that carbapenem and colistin resistance determinants are already circulating within the One Health interface. That why this paper info/evidence is more important for the scientific world. Similarly, colistin-resistant E. coli carrying **mcr** genes has been reported from poultry and poultry-associated environmental samples in Bangladesh. These findings suggest that resistance to critically important antimicrobials may emerge through environmental dissemination, horizontal gene transfer, and co-selection by other antimicrobial classes rather than direct exposure to carbapenems in poultry production.

In contrast, fluoroquinolone resistance generally requires specific target-site mutations and/or additional resistance mechanisms. Although the **parC** gene was detected in the sequenced isolate, the absence of additional mutations or other resistance mechanisms may explain the retained phenotypic susceptibility to ciprofloxacin, levofloxacin, and norfloxacin. We have added a discussion to clarify these points and emphasize that the molecular basis of carbapenem and colistin resistance warrants further investigation. [line 273-283]

5. Line 250: Better to use one term either antibiotic or antimicrobial and not both interchangeably because they mean different. Should be standardized throughout the manuscript for scientific consistency.

**Response:** Thank you for this valuable suggestion. We agree that the terms "antibiotic" and "antimicrobial" should be used consistently throughout the manuscript. Following the reviewer's recommendation, we have carefully revised the manuscript and standardized the terminology by using **"antimicrobial"** throughout the text for scientific consistency. [Yellow in color]

6. Table 2: This table is unnecessary otherwise use it to establish resistant pattern; that is how may isolates were resistant to which antibiotics, and their percentage frequencies, including MDR patterns.

**Response:** Thank you for this valuable suggestion. We agree that the original Table 2 did not effectively summarize the antimicrobial resistance patterns. Following the reviewer's recommendation, we have removed the original table and replaced it with a new figure (Figure 3), which provides a clearer visual presentation of the antimicrobial resistance data. The revised figure (figure 3) illustrates (i) the resistance profile of each P. multocida isolate against the tested antibiotics, (ii) the number and percentage of isolates resistant to each antimicrobial, and (iii) the multidrug resistance (MDR) patterns observed among the isolates. We believe this graphical presentation is more informative and allows readers to readily interpret the resistance frequencies and MDR profiles.

Reviewer #2: I do appreciate the authors for the wonderful study where they elucidated the genotypic and phenotypic resistance determinants of P. multocida isolated from Layer chicken in Bangladesh. Given the importance and devastation of Fowl cholera this study is of great importance which will guide developing control strategies. Besides, there are some limitations mentioned below which should be addressed to improve the scientific quality and acceptability of this manuscript.

1. The authors reported 12 type A strains, but WGS was done for only 1 isolate, which limits generalizability o this study. It’s not likely that all the isolates carries all the virulence gene described. For minimum representation, I would suggest performing WGS of at least 3 of the isolates.

**Response:** We sincerely thank the reviewer for this valuable suggestion. We fully agree that sequencing additional isolates would strengthen the conclusions and provide a more comprehensive understanding of the genomic diversity of P. multocida circulating in Bangladesh. Unfortunately, due to funding constraints and limited access to whole-genome sequencing facilities in our country, we were only able to sequence a single representative isolate. We have acknowledged this as a limitation in the Discussion section. The sequenced isolate was selected because it exhibited the broadest antimicrobial resistance profile among the confirmed isolates. We also recognize that the virulence gene profile identified from this isolate cannot be generalized to all 12 isolates. This study was conducted as part of a Master's research project with limited resources. We are actively seeking additional funding to sequence more isolates in our ongoing follow-up research, which will enable a more comprehensive comparative genomic analysis in the future. We appreciate the reviewer's constructive recommendation and have highlighted this limitation and future direction in the revised manuscript. [line 285-288]
2. The authors did WGS of a representative isolates PM58 which based on the Antibiotic resistance profile is resistant to Ampicillin, Cefixime, Ceftriaxone, Chloraphenicol, Colistin, Erythromycin, Ertapenem, Meropenem, Neomycin, Tetracycline. However, no corresponding resistance genes were shown from the ARGs analysis of the WGS.

**Response:** To address this concern, we reanalyzed the genome using dedicated antimicrobial resistance databases (NCBI AMRFinderPlus, CARD, and ResFinder). No acquired resistance genes corresponding to the observed multidrug-resistant phenotype were identified. This finding suggests that the resistance phenotype may instead result from chromosomal mutations, altered expression of intrinsic resistance determinants such as efflux systems, changes in membrane permeability, or other resistance mechanisms that are not currently represented in ARG databases. We have revised the manuscript to clarify this distinction. [Line 257-273]

3. Moreover, CRP is a regulatory protein, tufA is a housekeeping gene, uhpT mutations can contribute to fosfomycin resistance but presence of uphT alone is not proof of resistance. Similarly, parC requires specific mutations for fluoroquinolone resistance. However, no genetic evidence on specific mutation was described in the study. Taken together, phenotypic AST results are not correlating with the genotypic data.

Response: We thank the reviewer for this insightful comment. We agree that the presence of genes such as crp, tufA, uhpT, and parC alone should not be interpreted as evidence of antimicrobial resistance. In particular, resistance associated with parC and uhpT depends on specific nucleotide mutations or altered gene function rather than their mere presence. Likewise, crp is a global regulatory protein and tufA is a conserved housekeeping gene that are not antimicrobial resistance determinants.

The objective of our whole-genome analysis was to identify antimicrobial resistance genes and other genomic features associated with the isolate. A comprehensive analysis of chromosomal single nucleotide polymorphisms (SNPs) or resistance-associated mutations in housekeeping or regulatory genes was not included in the scope of this study. We have revised the manuscript to clarify that these genes are reported as antibiotic targets or resistance-associated loci described in the literature, rather than confirmed resistance determinants in PM58. We also acknowledge in the Discussion that future studies incorporating mutation analysis will be necessary to investigate genotype–phenotype discrepancies. To avoid any contradiction, we relocate that table as a supplementary file. [Line 257-273]

4. No experimental validation of virulence genes. Moreover, describing virulence genes like in Table 4 indicates authors poor knowledge on scientific presentation of genome analysis data. Similarly, Table 2 could be simplified. In current format, none of the Tables worth inclusion in the main text.

**Response:** We sincerely thank the reviewer for this constructive comment. We agree that experimental validation would provide stronger evidence for the biological roles of the identified virulence-associated genes. However, the objective of the present study was to characterize the genome of a representative P. multocida isolate through whole-genome sequencing and to identify putative virulence-associated genes using established bioinformatics databases. Functional validation of these genes was beyond the scope of the current study due to resource limitations. As this work represents the initial phase of an ongoing research project, we plan to perform additional sequencing, gene expression, and functional validation experiments in our subsequent studies to further investigate the roles of these virulence-associated genes.

Regarding the presentation of the virulence gene table, our intention was to provide readers with both the identified genes and their corresponding predicted products and reported biological functions in a single, easily interpretable format. We believe that presenting the functional information alongside the genomic findings improves the accessibility of the results, particularly for readers who may not specialize in bacterial genomics, and reduces the need to repeatedly consult the literature to understand the biological significance of each detected gene. Similar presentation formats have also been used in previous genome characterization studies. Therefore, we respectfully prefer to retain the current format, as we believe it enhances the clarity and interpretability of the genomic findings.

In addition, following the reviewer's suggestion regarding Table 2, we have substantially revised its presentation. The original antimicrobial susceptibility table has been replaced with a more informative graphical summary (figure 3) that illustrates the antimicrobial resistance frequencies, isolate-specific resistance patterns, and multidrug resistance (MDR) profiles, thereby improving the clarity and overall presentation of the antimicrobial susceptibility data.

5. I don’t understand the importance of Figure 4 in this manuscript. It doesn’t provide any information critical to this manuscript. Better to remove or move to Supplementary Figures.

**Response:** Thank you for this valuable suggestion. We agree that the circular genome map (Figure 4) is primarily descriptive and does not directly contribute to the main findings of the manuscript. Following the reviewer's recommendation, we have removed Figure 4.

6. Figure 5 showing distance tree confirmed species of bacteria, where the sequenced isolate is colored in yellow. Although the figure resolution is very poor, I could see the yellow highlighted name as 16S rRNA processing protein RimM [Pasteurella multocida]. I wonder, why? The isolate ID is not mathcing with the isolate ID or name described in the legend. Moreover, the tree is multiphylectic, I would like to know how the author ascertained the position of the isolate in the second monophyletic clade? And, this Figure upon proper justification can be moved to the Supplementary Figures.

**Response:** We thank the reviewer for this valuable comment. We agree that Figure 5 did not contribute substantially to the main findings of the manuscript. In response to the reviewer's suggestion, we have removed Figure 5 from the revised manuscript and added it as a supplementary file.

7. Figure 6 title is not scientifically connected to this manuscript. P. multocida is widely distributed and genomically connected. How this Title related to this study? Better to modify the title to represent a direct connection with this study.

**Response:** We thank the reviewer for this valuable comment. We agree that *Pasteurella multocida* is a globally distributed species with considerable genomic relatedness among strains. The purpose of Figure 6 was not to demonstrate the widespread distribution of the species but rather to place the Bangladeshi isolate BAUFCTA within the global genomic context and assess its evolutionary relationship with publicly available genomes. We have revised both the figure title to clarify this objective and avoid overstating the significance of the phylogenetic analysis.

8. Delete Fig. 7 and 8. Writing those data in text is enough. Rather, the author could perform other genomic analysis such as, mlst analysis, Capsular/LPS genotype confirmation from genome, plasmid analysis, MGEs, prophages, pan-genome comparisons which could provide deeper evolutionary and epidemiological insights.

**Response:** Thank you for this valuable suggestion. Following the reviewer's recommendation, Figures 7 and 8 have been removed from the main manuscript. We agree that additional genomic analyses would provide further evolutionary and epidemiological insights. However, these analyses were beyond the scope of the present study, which primarily aimed to report the first whole-genome sequence of a P. multocida type A isolate from Bangladeshi layer hens and to characterize its major virulence and some antimicrobial resistance determinants. We have acknowledged this as a limitation and included these analyses as important future research directions. Thank you.
