# Supplementary material for: Isolation, antibiogram and whole genome sequence analysis of Pasteurella multocida type A from layer birds
Source: PLoS One. 2026 Aug 31;21(8):e0357005. doi: 10.1371/journal.pone.0357005 (PMC13528996; doi:10.1371/journal.pone.0357005)
Supplement: Peer review history

# Peer review: Decision Letter 0

**DOI:** 10.1371/journal.pone.0357005.r001

**Authors:** Faham Khamesipour

4 Jun 2026

Dear Dr. Shahid,

Thank you for submitting your manuscript to PLOS ONE. After careful consideration, we feel that it has merit but does not fully meet PLOS ONE’s publication criteria as it currently stands. Therefore, we invite you to submit a revised version of the manuscript that addresses the points raised during the review process.

==============================

**ACADEMIC EDITOR:**

- The manuscript addresses an important and timely topic by investigating the antimicrobial susceptibility and whole-genome characteristics of *Pasteurella multocida*  type A isolates recovered from layer chickens in Bangladesh, and the reviewers acknowledge its potential contribution to poultry disease surveillance and antimicrobial resistance monitoring. However, several major concerns must be addressed before the manuscript can be considered for publication. The most significant limitation is that whole-genome sequencing was performed on only one isolate, while conclusions regarding virulence factors and antimicrobial resistance profiles are generalized to all isolates. In addition, substantial discrepancies exist between the phenotypic antimicrobial susceptibility results and the reported genomic resistance determinants, requiring a more rigorous analysis and interpretation of genotype–phenotype correlations. The presentation and interpretation of virulence genes should be revised to avoid overstatement of their biological significance, and additional genomic analyses (e.g., MLST, capsular/LPS typing, plasmid and mobile genetic element characterization) should be considered or their omission justified. Several figures and tables require substantial revision, simplification, or relocation to supplementary materials, and the rationale for antimicrobial selection, particularly chloramphenicol, carbapenems, and multiple quinolones, should be clearly explained. Furthermore, the reported resistance to carbapenems and colistin warrants additional discussion in the context of antimicrobial usage practices in Bangladesh. Clarification of methodological details, standardization of terminology, inclusion of appropriate statistical analyses where applicable, and careful revision of data presentation and interpretation are also necessary. Overall, while the study has merit and addresses an important veterinary and public health issue, a major revision is required to strengthen its scientific rigor and support the conclusions drawn.

Please submit your revised manuscript by Jul 19 2026 11:59PM. If you will need more time than this to complete your revisions, please reply to this message or contact the journal office at plosone@plos.org. When you're ready to submit your revision, log on to https://www.editorialmanager.com/pone/ and select the 'Submissions Needing Revision' folder to locate your manuscript file.

- A letter that responds to each point raised by the academic editor and reviewer(s). You should upload this letter as a separate file labeled 'Response to Reviewers'.
- A marked-up copy of your manuscript that highlights changes made to the original version. You should upload this as a separate file labeled 'Revised Manuscript with Track Changes'.
- An unmarked version of your revised paper without tracked changes. You should upload this as a separate file labeled 'Manuscript'.

If you would like to make changes to your financial disclosure, please include your updated statement in your cover letter. Guidelines for resubmitting your figure files are available below the reviewer comments at the end of this letter.

If applicable, we recommend that you deposit your laboratory protocols in protocols.io to enhance the reproducibility of your results. Protocols.io assigns your protocol its own identifier (DOI) so that it can be cited independently in the future. For instructions see: https://journals.plos.org/plosone/s/submission-guidelines#loc-laboratory-protocols. Additionally, PLOS ONE offers an option for publishing peer-reviewed Lab Protocol articles, which describe protocols hosted on protocols.io. Read more information on sharing protocols at https://plos.org/protocols?utm_medium=editorial-email&utm_source=authorletters&utm_campaign=protocols.

As the corresponding author, your ORCID iD is verified in the submission system and will appear in the published article. PLOS supports the use of ORCID, and we encourage all coauthors to register for an ORCID iD and use it as well. Please encourage your coauthors to verify their ORCID iD within the submission system before final acceptance, as unverified ORCID iDs will not appear in the published article. *Only*  the individual author can complete the verification step; PLOS staff *cannot*  verify ORCID iDs on behalf of authors.

We look forward to receiving your revised manuscript.

Kind regards,

Faham Khamesipour, Ph.D.

Academic Editor

PLOS One

Journal requirements:

When submitting your revision, we need you to address these additional requirements.

1. Please ensure that your manuscript meets PLOS ONE's style requirements, including those for file naming. The PLOS ONE style templates can be found at

https://journals.plos.org/plosone/s/file?id=wjVg/PLOSOne_formatting_sample_main_body.pdf and

https://journals.plos.org/plosone/s/file?id=ba62/PLOSOne_formatting_sample_title_authors_affiliations.pdf

2. In your Methods section, please provide additional information regarding the permits you obtained for the work. Please ensure you have included the full name of the authority that approved the field site access and, if no permits were required, a brief statement explaining why.

3. PLOS ONE now requires that authors provide the original uncropped and unadjusted images underlying all blot or gel results reported in a submission’s figures or Supporting Information files. This policy and the journal’s other requirements for blot/gel reporting and figure preparation are described in detail at https://journals.plos.org/plosone/s/figures#loc-blot-and-gel-reporting-requirements and https://journals.plos.org/plosone/s/figures#loc-preparing-figures-from-image-files. When you submit your revised manuscript, please ensure that your figures adhere fully to these guidelines and provide the original underlying images for all blot or gel data reported in your submission. See the following link for instructions on providing the original image data: https://journals.plos.org/plosone/s/figures#loc-original-images-for-blots-and-gels.

In your cover letter, please note whether your blot/gel image data are in Supporting Information or posted at a public data repository, provide the repository URL if relevant, and provide specific details as to which raw blot/gel images, if any, are not available. Email us at plosone@plos.org if you have any questions.

If the reviewer comments include a recommendation to cite specific previously published works, please review and evaluate these publications to determine whether they are relevant and should be cited. There is no requirement to cite these works unless the editor has indicated otherwise.

[Note: HTML markup is below. Please do not edit.]

Reviewers' comments:

Reviewer's Responses to Questions

**Comments to the Author**

1. Is the manuscript technically sound, and do the data support the conclusions?

Reviewer #1: Yes

Reviewer #2: No

**********

2. Has the statistical analysis been performed appropriately and rigorously?

Reviewer #1: N/A

Reviewer #2: No

**********

3. Have the authors made all data underlying the findings in their manuscript fully available?

The PLOS Data policy

Reviewer #1: Yes

Reviewer #2: Yes

**********

4. Is the manuscript presented in an intelligible fashion and written in standard English?

Reviewer #1: Yes

Reviewer #2: Yes

**********

Reviewer #1: Manuscript Title: Isolation, Antibiogram and Whole Genome Sequence Analysis of Pasteurella multocida Type A from Layer Birds

The manuscript presents important findings regarding the isolation, antimicrobial susceptibility, and whole-genome characterization of Pasteurella multocida type A isolated from layer birds in Bangladesh. The integration of phenotypic antimicrobial resistance testing with genomic analysis is valuable and relevant to poultry disease surveillance and antimicrobial resistance monitoring. However, several issues require clarification and revision before the manuscript can be considered for publication.

1. BDT: Please provide the long form in the first mention.

2. Line 94: 80-layer hens; from how many farms?

3. Line 139-145: Is chloramphenicol currently approved to be used in veterinary treatment in Bangladesh? If not, justification for its inclusion should be provided. Why testing three quinolones antibiotic discs? I can agree with cephalosporins because they are from different generations! Can you provide justification of using meropenem and ertapenem simultaneously. It is advised to use either of the guideline, otherwise please, state which guideline for which antibiotic discs.

4. Line 148: I'm very concerned with resistance of this pathogen to carbapenems and colistin while susceptible to Fluoroquinolones! Can you provide more explanation about this? Are carbapenems used in poultry production OR veterinary practices in Bangladesh?

5. Line 250: Better to use one term either antibiotic or antimicrobial and not both interchangeably because they mean different. Should be standardized throughout the manuscript for scientific consistency.

6. Table 2: This table is unnecessary otherwise use it to establish resistant pattern; that is how may isolates were resistant to which antibiotics, and their percentage frequencies, including MDR patterns.

Reviewer #2: I do appreciate the authors for the wonderful study where they elucidated the genotypic and phenotypic resistance determinants of P. multocida isolated from Layer chicken in Bangladesh. Given the importance and devastation of Fowl cholera this study is of great importance which will guide developing control strategies. Besides, there are some limitations mentioned below which should be addressed to improve the scientific quality and acceptability of this manuscript.

1. The authors reported 12 type A strains, but WGS was done for only 1 isolate, which limits generalizability o this study. It’s not likely that all the isolates carries all the virulence gene described. For minimum representation, I would suggest performing WGS of at least 3 of the isolates.

2. The authors did WGS of a representative isolates PM58 which based on the Antibiotic resistance profile is resistant to Ampicillin, Cefixime, Ceftriaxone, Chloraphenicol, Colistin, Erythromycin, Ertapenem, Meropenem, Neomycin, Tetracycline. However, no corresponding resistance genes were shown from the ARGs analysis of the WGS.

3. Moreover, CRP is a regulatory protein, tufA is a housekeeping gene, uhpT mutations can contribute to fosfomycin resistance but presence of uphT alone is not proof of resistance. Similarly, parC requires specific mutations for fluoroquinolone resistance. However, no genetic evidence on specific mutation was described in the study. Taken together, phenotypic AST results are not correlating with the genotypic data.

4. No experimental validation of virulence genes. Moreover, describing virulence genes like in Table 4 indicates authors poor knowledge on scientific presentation of genome analysis data. Similarly, Table 2 could be simplified. In current format, none of the Tables worth inclusion in the main text.

5. I don’t understand the importance of Figure 4 in this manuscript. It doesn’t provide any information critical to this manuscript. Better to remove or move to Supplementary Figures.

6. Figure 5 showing distance tree confirmed species of bacteria, where the sequenced isolate is colored in yellow. Although the figure resolution is very poor, I could see the yellow highlighted name as 16S rRNA processing protein RimM [Pasteurella multocida]. I wonder, why? The isolate ID is not mathcing with the isolate ID or name described in the legend. Moreover, the tree is multiphylectic, I would like to know how the author ascertained the position of the isolate in the second monophyletic clade? And, this Figure upon proper justification can be moved to the Supplementary Figures.

7. Figure 6 title is not scientifically connected to this manuscript. P. multocida is widely distributed and genomically connected. How this Title related to this study? Better to modify the title to represent a direct connection with this study.

8. Delete Fig. 7 and 8. Writing those data in text is enough. Rather, the author could perform other genomic analysis such as, mlst analysis, Capsular/LPS genotype confirmation from genome, plasmid analysis, MGEs, prophages, pan-genome comparisons which could provide deeper evolutionary and epidemiological insights.

9. The manuscript reported descriptive results, and no statistical analysis was evident.

Overall, the study is novel but sequencing only one isolate, overestimation of the virulence and resistance profiles, the authors knowledge on analyzing and presenting genomic data downgraded the quality. Thus, claims on the virulence and AMR should be toned down, and the suggested modification would help upholding the quality and scientific rigor of this study.

**********

what does this mean?). If published, this will include your full peer review and any attached files.

If you choose “no”, your identity will remain anonymous but your review may still be made public.

**Do you want your identity to be public for this peer review?**  For information about this choice, including consent withdrawal, please see our Privacy Policy

Reviewer #1: No

Reviewer #2: **Yes:** Jayedul Hassan

**********

[NOTE: If reviewer comments were submitted as an attachment file, they will be attached to this email and accessible via the submission site. Please log into your account, locate the manuscript record, and check for the action link "View Attachments". If this link does not appear, there are no attachment files.]

To ensure your figures meet our technical requirements, please review our figure guidelines: https://journals.plos.org/plosone/s/figures

You may also use PLOS’s free figure tool, NAAS, to help you prepare publication quality figures: https://journals.plos.org/plosone/s/figures#loc-tools-for-figure-preparation.

NAAS will assess whether your figures meet our technical requirements by comparing each figure against our figure specifications.

# Author comment: Author response to Decision Letter 1

**DOI:** 10.1371/journal.pone.0357005.r002

24 Jul 2026

The manuscript addresses an important and timely topic by investigating the antimicrobial susceptibility and whole-genome characteristics of Pasteurella multocida type A isolates recovered from layer chickens in Bangladesh, and the reviewers acknowledge its potential contribution to poultry disease surveillance and antimicrobial resistance monitoring. However, several major concerns must be addressed before the manuscript can be considered for publication.

The most significant limitation is that whole-genome sequencing was performed on only one isolate, while conclusions regarding virulence factors and antimicrobial resistance profiles are generalized to all isolates. In addition, substantial discrepancies exist between the phenotypic antimicrobial susceptibility results and the reported genomic resistance determinants, requiring a more rigorous analysis and interpretation of genotype–phenotype correlations. The presentation and interpretation of virulence genes should be revised to avoid overstatement of their biological significance, and additional genomic analyses (e.g., MLST, capsular/LPS typing, plasmid and mobile genetic element characterization) should be considered or their omission justified. Several figures and tables require substantial revision, simplification, or relocation to supplementary materials, and the rationale for antimicrobial selection, particularly chloramphenicol, carbapenems, and multiple quinolones, should be clearly explained. Furthermore, the reported resistance to carbapenems and colistin warrants additional discussion in the context of antimicrobial usage practices in Bangladesh. Clarification of methodological details, standardization of terminology, inclusion of appropriate statistical analyses where applicable, and careful revision of data presentation and interpretation are also necessary. Overall, while the study has merit and addresses an important veterinary and public health issue, a major revision is required to strengthen its scientific rigor and support the conclusions drawn.

Response: We sincerely thank the Editor for the careful evaluation of our manuscript and for the constructive summary of the major concerns. We appreciate the opportunity to revise our work and have carefully addressed all comments raised by both the Editor and the reviewers. The manuscript has been substantially revised to improve its scientific rigor, clarity, and presentation.

Specifically, we have acknowledged that whole-genome sequencing was performed on a single representative isolate and have clearly stated this as a limitation of the study. We emphasize that the identified virulence-associated genes and genomic characteristics are specific to the sequenced isolate and should not be generalized to all P. multocida isolates. We have also highlighted that additional sequencing and comparative genomic analyses are planned in our ongoing research.

To address the discrepancy between phenotypic antimicrobial susceptibility and genomic resistance determinants, we reanalyzed the genome using three dedicated antimicrobial resistance databases (NCBI AMRFinderPlus, CARD, and ResFinder). No acquired resistance genes corresponding to the observed multidrug-resistant phenotype were identified. Accordingly, we revised both the Results and Discussion to clarify that the observed resistance may result from chromosomal mutations, altered expression of intrinsic resistance mechanisms, changes in membrane permeability, efflux systems, or other mechanisms not represented in current resistance gene databases. We also revised the interpretation of crp, tufA, uhpT, and parC, making it clear that these genes are not, by themselves, confirmed antimicrobial resistance determinants. Also the table has been removed from the main file.

The presentation and interpretation of virulence-associated genes have also been revised to avoid overstatement. We clearly state that these genes were identified through in silico genome annotation and have not been experimentally validated. This limitation has been acknowledged in the manuscript, and future studies involving gene expression and functional validation have been proposed.

The manuscript presentation has been substantially improved. The original antimicrobial susceptibility table has been replaced with a graphical summary illustrating antimicrobial resistance frequencies, isolate-specific resistance patterns, and multidrug resistance (MDR) profiles. Descriptive genome figures have been removed from the main manuscript or transferred to the Supplementary Materials where appropriate. Figure titles, legends, and phylogenetic descriptions have also been revised to better reflect the scope of the analyses.

We have clarified the rationale for the antimicrobial panel used in this study, including the inclusion of chloramphenicol, carbapenems, and multiple fluoroquinolones, emphasizing that these agents were included for epidemiological surveillance and comparison with previous studies rather than as therapeutic recommendations. Furthermore, the Discussion has been expanded to explain the observed resistance to carbapenems and colistin in the context of antimicrobial resistance reports from Bangladesh and the broader One Health framework.

Finally, we carefully revised the manuscript for language, terminology, methodological clarity, and scientific consistency. The terminology has been standardized by consistently using "antimicrobial" throughout the manuscript where appropriate, methodological details have been clarified, figure and table presentation has been improved, and the overall discussion has been revised to provide a more balanced interpretation of the findings.

We believe these revisions have substantially strengthened the manuscript and have addressed the concerns raised by both the Editor and the reviewers. We sincerely appreciate the time and effort devoted to evaluating our work and hope that the revised manuscript is now suitable for publication.

Reviewer #1:

Manuscript Title: Isolation, Antibiogram and Whole Genome Sequence Analysis of Pasteurella multocida Type A from Layer Birds

The manuscript presents important findings regarding the isolation, antimicrobial susceptibility, and whole-genome characterization of Pasteurella multocida type A isolated from layer birds in Bangladesh. The integration of phenotypic antimicrobial resistance testing with genomic analysis is valuable and relevant to poultry disease surveillance and antimicrobial resistance monitoring. However, several issues require clarification and revision before the manuscript can be considered for publication.

1. BDT: Please provide the long form in the first mention.

Instead of using short form we used now in full form of BDT.

Response: Thanks for the comment and carefully addressed the issue. [line 49]

2. Line 94: 80-layer hens; from how many farms?

Response: Thanks for asking but farm numbers are already mentioned in line 87-89.

3. Line 139-145: Is chloramphenicol currently approved to be used in veterinary treatment in Bangladesh? If not, justification for its inclusion should be provided. Why testing three quinolones antibiotic discs? I can agree with cephalosporins because they are from different generations! Can you provide justification of using meropenem and ertapenem simultaneously.

Response: Thank you for this important comment. We clarify that this panel was selected for AMR surveillance and comparison with previous regional studies, not as a therapeutic recommendation for poultry. We acknowledge that some antibiotics included in the panel, such as chloramphenicol, meropenem, and ertapenem, are not intended or recommended for use in food-producing animals. Therefore, we have revised the manuscript to clearly state that results for these antibiotics should be interpreted only for epidemiological and One Health AMR monitoring purposes.

Regarding the quinolones/fluoroquinolones, ciprofloxacin, enrofloxacin, levofloxacin, and norfloxacin were included because these drugs are commonly reported in AMR studies and allow comparison of susceptibility patterns within this antimicrobial class. Enrofloxacin is particularly relevant to veterinary use, whereas ciprofloxacin, levofloxacin, and norfloxacin provide comparative surveillance information.

Meropenem and ertapenem were included together to assess whether resistance was consistent across carbapenem agents. However, we agree that these results should not imply clinical use in poultry. We have therefore added a clarification that these drugs were included only for resistance surveillance at the end of discussion. [Line 273-283]

4. Line 148: I'm very concerned with resistance of this pathogen to carbapenems and colistin while susceptible to Fluoroquinolones! Can you provide more explanation about this? Are carbapenems used in poultry production OR veterinary practices in Bangladesh?

Response: Thank you for this important comment. We agree that the observed resistance pattern deserves further discussion. To our knowledge, carbapenems are not approved or routinely used in poultry production or veterinary practice in Bangladesh. Therefore, the observed carbapenem resistance is unlikely to result from direct therapeutic use in poultry. However, most recent studies from Bangladesh (10.1128/spectrum.03956-23, 10.5455/javar.2019.f311, 10.1038/s41598-020-75608-2) have reported colistin and carbapenem-resistant microbe in poultry, humans, and poultry-associated environments, suggesting that carbapenem and colistin resistance determinants are already circulating within the One Health interface. That why this paper info/evidence is more important for the scientific world. Similarly, colistin-resistant E. coli carrying mcr genes has been reported from poultry and poultry-associated environmental samples in Bangladesh. These findings suggest that resistance to critically important antimicrobials may emerge through environmental dissemination, horizontal gene transfer, and co-selection by other antimicrobial classes rather than direct exposure to carbapenems in poultry production.

In contrast, fluoroquinolone resistance generally requires specific target-site mutations and/or additional resistance mechanisms. Although the parC gene was detected in the sequenced isolate, the absence of additional mutations or other resistance mechanisms may explain the retained phenotypic susceptibility to ciprofloxacin, levofloxacin, and norfloxacin. We have added a discussion to clarify these points and emphasize that the molecular basis of carbapenem and colistin resistance warrants further investigation. [line 273-283]

5. Line 250: Better to use one term either antibiotic or antimicrobial and not both interchangeably because they mean different. Should be standardized throughout the manuscript for scientific consistency.

Response: Thank you for this valuable suggestion. We agree that the terms "antibiotic" and "antimicrobial" should be used consistently throughout the manuscript. Following the reviewer's recommendation, we have carefully revised the manuscript and standardized the terminology by using "antimicrobial" throughout the text for scientific consistency. [Yellow in color]

6. Table 2: This table is unnecessary otherwise use it to establish resistant pattern; that is how may isolates were resistant to which antibiotics, and their percentage frequencies, including MDR patterns.

Response: Thank you for this valuable suggestion. We agree that the original Table 2 did not effectively summarize the antimicrobial resistance patterns. Following the reviewer's recommendation, we have removed the original table and replaced it with a new figure (Figure 3), which provides a clearer visual presentation of the antimicrobial resistance data. The revised figure (figure 3) illustrates (i) the resistance profile of each P. multocida isolate against the tested antibiotics, (ii) the number and percentage of isolates resistant to each antimicrobial, and (iii) the multidrug resistance (MDR) patterns observed among the isolates. We believe this graphical presentation is more informative and allows readers to readily interpret the resistance frequencies and MDR profiles.

Reviewer #2: I do appreciate the authors for the wonderful study where they elucidated the genotypic and phenotypic resistance determinants of P. multocida isolated from Layer chicken in Bangladesh. Given the importance and devastation of Fowl cholera this study is of great importance which will guide developing control strategies. Besides, there are some limitations mentioned below which should be addressed to improve the scientific quality and acceptability of this manuscript.

1. The authors reported 12 type A strains, but WGS was done for only 1 isolate, which limits generalizability o this study. It’s not likely that all the isolates carries all the virulence gene described. For minimum representation, I would suggest performing WGS of at least 3 of the isolates.

Response: We sincerely thank the reviewer for this valuable suggestion. We fully agree that sequencing additional isolates would strengthen the conclusions and provide a more comprehensive understanding of the genomic diversity of P. multocida circulating in Bangladesh. Unfortunately, due to funding constraints and limited access to whole-genome sequencing facilities in our country, we were only able to sequence a single representative isolate. We have acknowledged this as a limitation in the Discussion section. The sequenced isolate was selected because it exhibited the broadest antimicrobial resistance profile among the confirmed isolates. We also recognize that the virulence gene profile identified from this isolate cannot be generalized to all 12 isolates. This study was conducted as part of a Master's research project with limited resources. We are actively seeking additional funding to sequence more isolates in our ongoing follow-up research, which will enable a more comprehensive comparative genomic analysis in the future. We appreciate the reviewer's constructive recommendation and have highlighted this limitation and future direction in the revised manuscript. [line 285-288]

2. The authors did WGS of a representative isolates PM58 which based on the Antibiotic resistance profile is resistant to Ampicillin, Cefixime, Ceftriaxone, Chloraphenicol, Colistin, Erythromycin, Ertapenem, Meropenem, Neomycin, Tetracycline. However, no corresponding resistance genes were shown from the ARGs analysis of the WGS.

Response: To address this concern, we reanalyzed the genome using dedicated antimicrobial resistance databases (NCBI AMRFinderPlus, CARD, and ResFinder). No acquired resistance genes corresponding to the observed multidrug-resistant phenotype were identified. This finding suggests that the resistance phenotype may instead result from chromosomal mutations, altered expression of intrinsic resistance determinants such as efflux systems, changes in membrane permeability, or other resistance mechanisms that are not currently represented in ARG databases. We have revised the manuscript to clarify this distinction. [Line 257-273]

3. Moreover, CRP is a regulatory protein, tufA is a housekeeping gene, uhpT mutations can contribute to fosfomycin resistance but presence of uphT alone is not proof of resistance. Similarly, parC requires specific mutations for fluoroquinolone resistance. However, no genetic evidence on specific mutation was described in the study. Taken together, phenotypic AST results are not correlating with the genotypic data.

Response: We thank the reviewer for this insightful comment. We agree that the presence of genes such as crp, tufA, uhpT, and parC alone should not be interpreted as evidence of antimicrobial resistance. In particular, resistance associated with parC and uhpT depends on specific nucleotide mutations or altered gene function rather than their mere presence. Likewise, crp is a global regulatory protein and tufA is a conserved housekeeping gene that are not antimicrobial resistance determinants.

The objective of our whole-genome analysis was to identify antimicrobial resistance genes and other genomic features associated with the isolate. A comprehensive analysis of chromosomal single nucleo

Attachment Submitted filename: Response to Reviewers.docx

# Peer review: Decision Letter 1

**DOI:** 10.1371/journal.pone.0357005.r003

**Authors:** Faham Khamesipour

12 Aug 2026

Isolation, Antibiogram and Whole Genome Sequence Analysis of Pasteurella multocida Type A from Layer Birds

PONE-D-26-21400R1

Dear Dr. Shahid,

We’re pleased to inform you that your manuscript has been judged scientifically suitable for publication and will be formally accepted for publication once it meets all outstanding technical requirements.

Within one week, you’ll receive an e-mail detailing the required amendments. When these have been addressed, you’ll receive a formal acceptance letter and your manuscript will be scheduled for publication.

An invoice will be generated when your article is formally accepted. Please note, if your institution has a publishing partnership with PLOS and your article meets the relevant criteria, all or part of your publication costs will be covered. Please make sure your user information is up-to-date by logging into Editorial Manager at Editorial Manager® and clicking the ‘Update My Information' link at the top of the page. For questions related to billing, please contact billing support.

If your institution or institutions have a press office, please notify them about your upcoming paper to help maximize its impact. If they’ll be preparing press materials, please inform our press team as soon as possible -- no later than 48 hours after receiving the formal acceptance. Your manuscript will remain under strict press embargo until 2 pm Eastern Time on the date of publication. For more information, please contact onepress@plos.org.

Kind regards,

Faham Khamesipour, Ph.D.

Academic Editor

PLOS One

Additional Editor Comments (optional):

Reviewers' comments:

Reviewer's Responses to Questions

**Comments to the Author**

Reviewer #1: All comments have been addressed

Reviewer #3: All comments have been addressed

**********

2. Is the manuscript technically sound, and do the data support the conclusions?

Reviewer #1: Yes

Reviewer #3: Yes

**********

3. Has the statistical analysis been performed appropriately and rigorously?

Reviewer #1: N/A

Reviewer #3: N/A

**********

4. Have the authors made all data underlying the findings in their manuscript fully available?

The PLOS Data policy

Reviewer #1: Yes

Reviewer #3: Yes

**********

5. Is the manuscript presented in an intelligible fashion and written in standard English?

Reviewer #1: Yes

Reviewer #3: Yes

**********

Reviewer #1: Next time do not lump several antimicrobials from one class, it is wastage of resources. Selection of antimicrobials should be based on class representation of the bacterium and epidemiological information of the study area. It is important to mention what proportion of isolates that were MDR. If it 100% as it is seen in the profile, say it in the text. Remember to define what MDR mean in your context.

Reviewer #3: Thankyou for taking the time to revise the manuscript. It is clear that alot of work has gone into improving this version of the manuscript. The limitations are clearly outlined and the evidence provided to support each of the sections, even more than I might regularly see in manuscripts, so thankyou. In the future though, please ensure to include the rationale and calculation underpinning the sample size (number of birds) collected so that calculations of prevalence are more robust.

**********

what does this mean?). If published, this will include your full peer review and any attached files.

If you choose “no”, your identity will remain anonymous but your review may still be made public.

**Do you want your identity to be public for this peer review?**  For information about this choice, including consent withdrawal, please see our Privacy Policy

Reviewer #1: **Yes:** Alexanda Mzula

Reviewer #3: No

**********

# Editor report: Acceptance letter

**DOI:** 10.1371/journal.pone.0357005.r004

**Authors:** Faham Khamesipour

PONE-D-26-21400R1

PLOS One

Dear Dr. Shahid,

I'm pleased to inform you that your manuscript has been deemed suitable for publication in PLOS One. Congratulations! Your manuscript is now being handed over to our production team.

At this stage, our production department will prepare your paper for publication. This includes ensuring the following:

* All references, tables, and figures are properly cited

* All relevant supporting information is included in the manuscript submission,

* There are no issues that prevent the paper from being properly typeset

You will receive further instructions from the production team, including instructions on how to review your proof when it is ready. Please keep in mind that we are working through a large volume of accepted articles, so please give us a few days to review your paper and let you know the next and final steps.

Lastly, if your institution or institutions have a press office, please let them know about your upcoming paper now to help maximize its impact. If they'll be preparing press materials, please inform our press team within the next 48 hours. Your manuscript will remain under strict press embargo until 2 pm Eastern Time on the date of publication. For more information, please contact onepress@plos.org.

You will receive an invoice from PLOS for your publication fee after your manuscript has reached the completed accept phase. If you receive an email requesting payment before acceptance or for any other service, this may be a phishing scheme. Learn how to identify phishing emails and protect your accounts at https://explore.plos.org/phishing.

If we can help with anything else, please email us at customercare@plos.org.

Thank you for submitting your work to PLOS One and supporting open access.

Kind regards,

PLOS ONE Editorial Office Staff

on behalf of

Dr. Faham Khamesipour

Academic Editor

PLOS One
